# An Integrated Metabolomic and Microbiome Analysis Identified Specific Gut Microbiota Associated with Fecal Cholesterol and Coprostanol in *Clostridium difficile* Infection

**DOI:** 10.1371/journal.pone.0148824

**Published:** 2016-02-12

**Authors:** Vijay C. Antharam, Daniel C. McEwen, Timothy J. Garrett, Aaron T. Dossey, Eric C. Li, Andrew N. Kozlov, Zhubene Mesbah, Gary P. Wang

**Affiliations:** 1 Department of Medicine, Division of Infectious Diseases and Global Medicine, University of Florida, Gainesville, FL, United States of America; 2 Department of Biosciences, Minnesota State University Moorhead, Moorhead, MN, United States of America; 3 Department of Pathology, Immunology, and Laboratory Medicine, University of Florida, Gainesville, FL, United States of America; 4 All Things Bugs LLC, Athens, GA, United States of America; 5 Medical Service, North Florida/South Georgia Veterans Health System, Gainesville, FL, United States of America; Institute Pasteur, FRANCE

## Abstract

*Clostridium difficile* infection (CDI) is characterized by dysbiosis of the intestinal microbiota and a profound derangement in the fecal metabolome. However, the contribution of specific gut microbes to fecal metabolites in *C*. *difficile*-associated gut microbiome remains poorly understood. Using gas-chromatography mass spectrometry (GC-MS) and 16S rRNA deep sequencing, we analyzed the metabolome and microbiome of fecal samples obtained longitudinally from subjects with *Clostridium difficile* infection (n = 7) and healthy controls (n = 6). From 155 fecal metabolites, we identified two sterol metabolites at >95% match to cholesterol and coprostanol that significantly discriminated *C*. *difficile-*associated gut microbiome from healthy microbiota. By correlating the levels of cholesterol and coprostanol in fecal extracts with 2,395 bacterial operational taxonomic units (OTUs) determined by 16S rRNA sequencing, we identified 63 OTUs associated with high levels of coprostanol and 2 OTUs correlated with low coprostanol levels. Using indicator species analysis (ISA), 31 of the 63 coprostanol-associated bacteria correlated with health, and two *Veillonella* species were associated with low coprostanol levels that correlated strongly with CDI. These 65 bacterial taxa could be clustered into 12 sub-communities, with each community containing a consortium of organisms that co-occurred with one another. Our studies identified 63 human gut microbes associated with cholesterol-reducing activities. Given the importance of gut bacteria in reducing and eliminating cholesterol from the GI tract, these results support the recent finding that gut microbiome may play an important role in host lipid metabolism.

## Introduction

The known microbial community imbalance associated with *Clostridium difficile* infection (CDI) [1–8] also implies disrupted metabolic profiles. Restoration of colonic microbiota is one of the most effective approaches for the treatment of CDI, which affects nearly half a million individuals per year in the US [9]. Since the gut microbiome of patients with CDI is significantly different from that of healthy individuals [2], differences in microbial composition is likely accompanied by alterations in fecal metabolites that define these two populations. Given the known depletion of gut microbiota in CDI, we hypothesized that an integrative analysis of fecal metabolome and microbiome would lead to the identification of fecal metabolites associated with specific gut microbes.

Using a gas chromatography-mass spectrometry (GC-MS) based fecal metabolomics approach; we observed that the levels of cholesterol and its reduced metabolite coprostanol in fecal samples were significantly different between CDI and healthy controls. Previous studies in gut physiology have established a role for gut bacteria in cholesterol metabolism. Such microorganisms were first described in 1934 [10, 11] and later identified as constituents of the human intestinal microbiota [12–14]. Given their cholesterol-reducing activity, these microbes have been investigated as potential agents for the treatment of hypercholesterolemia [15] and as additives to dairy products [16]. Cholesterol comprises up to 20% of the metabolites in fecal matter and their byproducts such as coprostanol and cholestanone contribute to an additional 5% of neutral sterol material [17].

Certain bacteria enzymatically reduce the double bond between carbons 5–6 of cholesterol to coprostanol, a reduced sterol, which is excreted in feces. It has been suggested that a high efficiency of cholesterol to coprostanol metabolism may reduce the risk of cardiovascular disease [18]. When coprostanol is conjugated with oligosaccharides, the resulting compounds have shown some activity against certain cancers [19, 20]. Low rates of cholesterol to coprostanol conversion have been implicated in the progression of ulcerative colitis [21, 22] and colon cancer [17]. Cholesterol reduction by microbiota can be achieved by bile-salt hydrolase (BSH) activity, binding to cell walls, enzymatic deconjugation, or direct uptake by the host bacteria [23, 24]. In *in-vitro* culture assays, certain strains of *Lactobacillus*, *Bifidobacterium*, *Enterococcus*, and *Streptococcus* have all shown to decrease the level of cholesterol [16, 24]. Together, the available data suggest a role for gut microbiota in fecal sterol metabolism. However, the identity of human endogenous gut microbes associated with cholesterol reduction remains poorly understood.

Here, we determined and measured cholesterol and coprostanol levels in fecal samples using GS-MS fecal metabolomics and found that levels of these two fecal metabolites differed significantly between subjects with CDI and healthy controls. Using multivariate Spearman rank correlation and 16S rRNA deep sequencing, we identified 65 bacterial phylotypes that were significantly associated with cholesterol or coprostanol, which included 63 phylotypes that correlated strongly with high coprostanol levels. Functional analysis of these 65 bacteria identified here would be of great interest for future studies.

## Results

### Fecal coprostanol and cholesterol levels in fecal samples distinguished CDI from healthy controls

**Fig 1:**
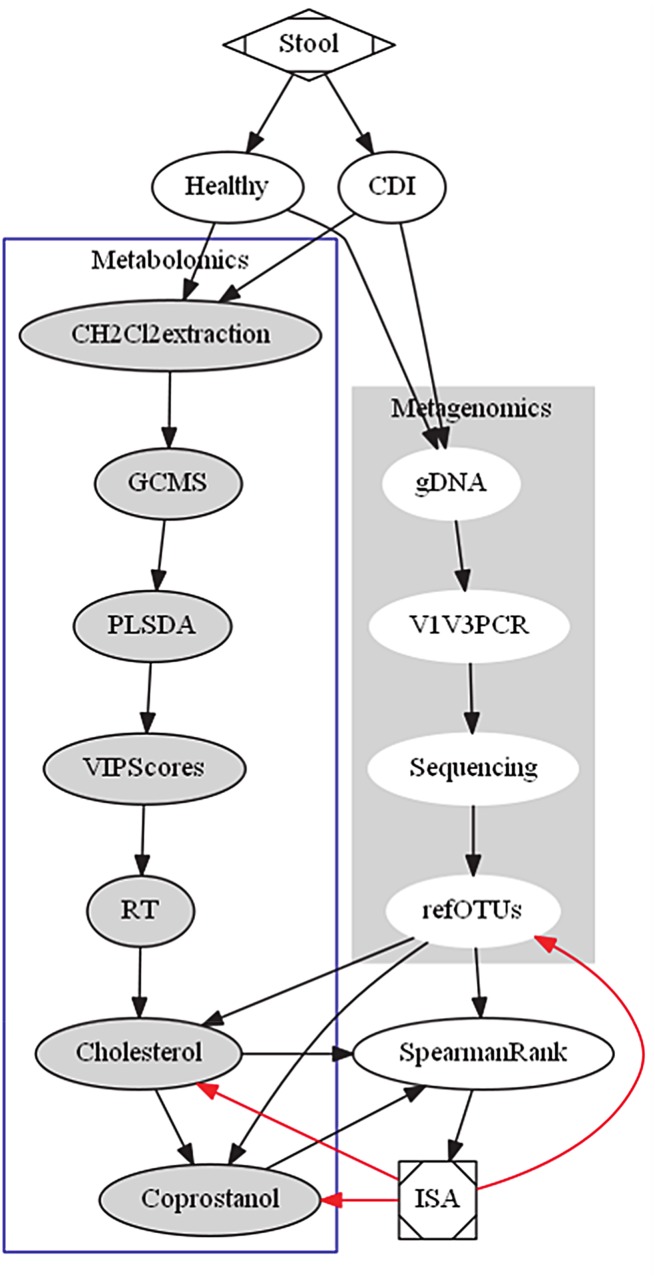
Flow-chart of integrative scheme between genomics and metabolomics to identify bacterial OTUs associated with cholesterol and coprostanol. Genomic DNA (gDNA) from longitudinal fecal samples emanating from Healthy or CDI subjects (over 90 days) was isolated and deep sequenced on the V1V3 hypervariable 16s rRNA gene before being classified to 2395 refOTUs (Right). The same longitudinal fecal sample was extracted with dichloromethane and injected on a GC-MS instrument where the retention time of discriminatory peaks were determined based on PLS-DA VIP scores (Left). Discriminatory peaks cholesterol and coprostanol were Spearman correlated to refOTUs based on NMDS and ANOVA. As a further step, ISA was used to determine whether refOTUs associated with high coprostanol or cholesterol were enriched in Healthy or CDI cohorts. Red arrows represent feedback and integration between chart items whereas black arrows are directional flow of the pipeline. Abbreviations: ANOVA: analysis of variance, ISA: indicator species analysis, NMDS: non-metric multidimensional scaling, PLS-DA: partial least squares discriminant analysis refOTUs: reference operational taxonomic units, RT: retention time, VIP scores: Variable importance in projection scores, CH2Cl2: dichloromethane.

**Fig 2:**
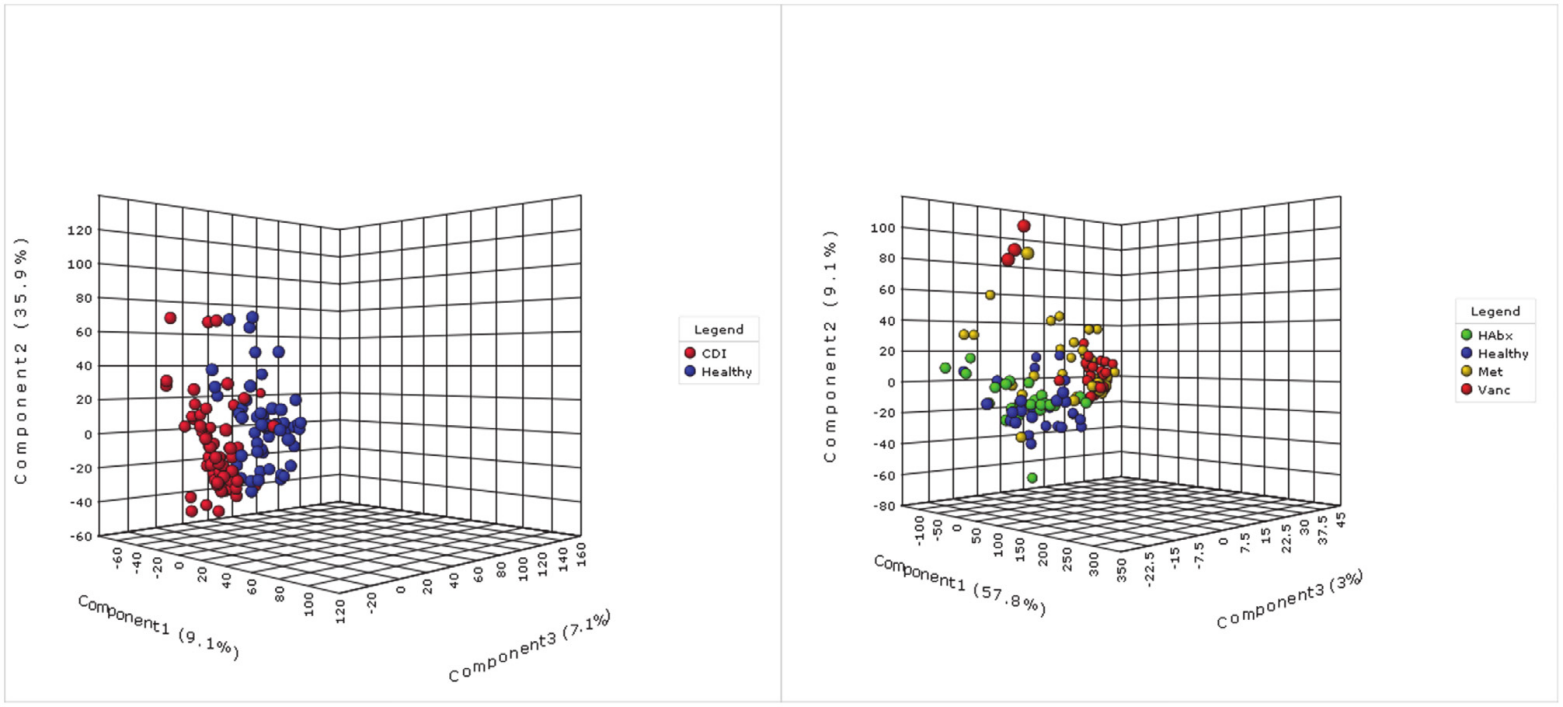
PLS-DA plots using a (left) two-state model, and (right) 4-state model. Antibiotic therapy for CDI (Met: Metronidazole, Vanc: Vancomycin) and antibiotic exposure history (HAbx: antibiotic exposure; Healthy: no antibiotic exposure) were used to distinguish groups. A matrix of retention time intensities were sum normalized and auto-scaled to generate both plots using a metabolomics pipeline established by Xia, et al. Each sphere represents a fecal chromatographic sample.

To identify fecal metabolites associated with specific gut microbes, we devised an integrative approach to correlate GC-MS metabolomics and 16S rRNA microbiome datasets (Fig 1). First, we examined metabolomics profiles of all samples collected longitudinally from seven subjects with CDI and six healthy controls (Table 1). Partial least squares-discriminant analysis (PLS-DA) showed a clear separation of metabolomics datasets between CDI and healthy controls, with 72.7% of the variation explained in three components (Fig 2A). The cross-validated predictive ability Q2 was 0.66, indicating that a random fecal GC-MS spectrum discriminates CDI from healthy controls at 66% of the time. The explained variance R2 was 0.88. We next divided the CDI cohort according to the antimicrobial treatment they received (either Metronidazole or Vancomycin), and the healthy controls according to their history of antibiotic exposure (HAbx: presence of recent antibiotic exposure, Healthy: absence of recent antibiotic exposure). PLS-DA using a 4-state model (Healthy, HAbx, Met, and Vanc) showed that 59.5% of the variation in the GC-MS dataset was explained (Fig 2B), with Q2 of 0.57 and R2 of 0.86 for a typical chromatogram. Thus, these results indicate distinct clustering of fecal metabolome between groups.

**Table 1:** Subject characteristics used in this study. The number of fecal samples analyzed is shown in parenthesis.

Group	Subject	Age	Sex	Prior Abx *	Concurrent Abx **	Prior history of CDI	Severity of CDI	CDI outcome^¶^
Met	101 (8)	68	Female	None	Cefazolin	No	Severe	Cure
Met	102 (10)	57	Female	Ciprofloxacin	Levofloxacin, Aztreonam	No	Moderate	Cure
Met	103 (6)	53	Female	Yes, with unknown Abx	Cefepime, Levofloxacin, TMP/SMX ***	Yes	Mild	Cure
Met	104 (10)	54	Female	Cefepime, IV vancomycin	Cefepime, IV Vancomycin	No	Mild-moderate	Recurrence
Vanc	301 (8)	44	Female	PO vancomycin	None	Yes	Severe	Cure
Vanc	302 (10)	28	Male	None	Cefepime, Linezolid, Ceftriaxone, IV Vancomycin	No	Severe	Cure
Vanc	303 (10)	27	Female	Amoxicillin	Ciprofloxacin, Levofloxacin	No	Severe	Cure
Habx	501 (7)	29	Female	Amoxicillin	None	No	N/A	N/A
Habx	502 (8)	60	Female	Doxycycline	None	No	N/A	N/A
Habx	503 (10)	45	Male	IV vancomycin	Cefepime, TMP/SMX ***	No	N/A	N/A
Healthy	701 (10)	21	Male	None	None	No	N/A	N/A
Healthy	702 (7)	20	Male	None	None	No	N/A	N/A
Healthy	703 (9)	53	Male	None	None	No	N/A	N/A

* Prior Abx: Antibiotics received during the 90 days prior to the date of the first sample collection

** Concurrent Abx: antibiotics (other than metronidazole or oral vancomycin for the treatment of CDI) received during the 90-day study period when stool samples were collected

*** TMP/SMX: Trimethoprim/Sulfamethoxazole

^¶^ Within 90 days of the study

To determine differentially abundant metabolites, Variable Importance Projection (VIP) scores from 114 fecal longitudinal chromatograms (S1 Fig) were generated for all longitudinal subject participants. As part of the metabolomics pipeline [25, 26], each retention time RT was assigned a VIP score for all components fitted to a PLS-DA model. We summed the contributions of each RT to the first three components (component 1, component 2, and component 3) which corresponds to the x, y, and z-axis in Fig 2. The highest ranked VIP scores were in the range of 31.900 ± 0.150 and 31.700 ± 0.150 minutes, which represented attractive targets for further identification by mass spectrometry (Part A in S2 Fig). Peaks corresponding to retention times identified as cholesterol showed clustering in regards to its contributions to the PLS-DA plot. Fecal coprostanol was identified in both Healthy, HAbx, and Metronidazole cohorts. Their chromatographic peaks in terms of retention times varied within a time window as shown in mapping the VIP scores for each cohort. Scattering of retention times identified as coprostanol for each fecal sample is reflected by the dispersion shown in the contribution of coprostanol towards its PLS-DA contribution (Part B in S2 Fig). In general, coprostanol and cholesterol peak RTs were identified within a range of ±0.150 min as stated above.

As a second method to identify discriminating RTs for subsequent analysis, we examined a two-group model (CDI vs Healthy), of which the lowest p-value from a t-test was observed at 31.927 minutes (mins) (p = 9.40 x 10^−6^) for the CDI group and 31.681 mins (p = 1.40 x 10^−11^) for the Healthy group. For a 4-group model (Met vs Vanc vs Healthy vs HAbx), the lowest p-value from ANOVA was observed at 31.843 mins (p = 7.25 x 10^−16^) for the HAbx cohort and 32.05 mins (p = 7.25 x 10^−13^) for the Vancomycin cohort. Thus, using a combination of PLS-DA VIP scores, t-test, and ANOVA (when stratifying Healthy and CDI), we identified a range of retention times that represented the most attractive targets for chemical identification by mass spectrometry.

**Fig 3:**
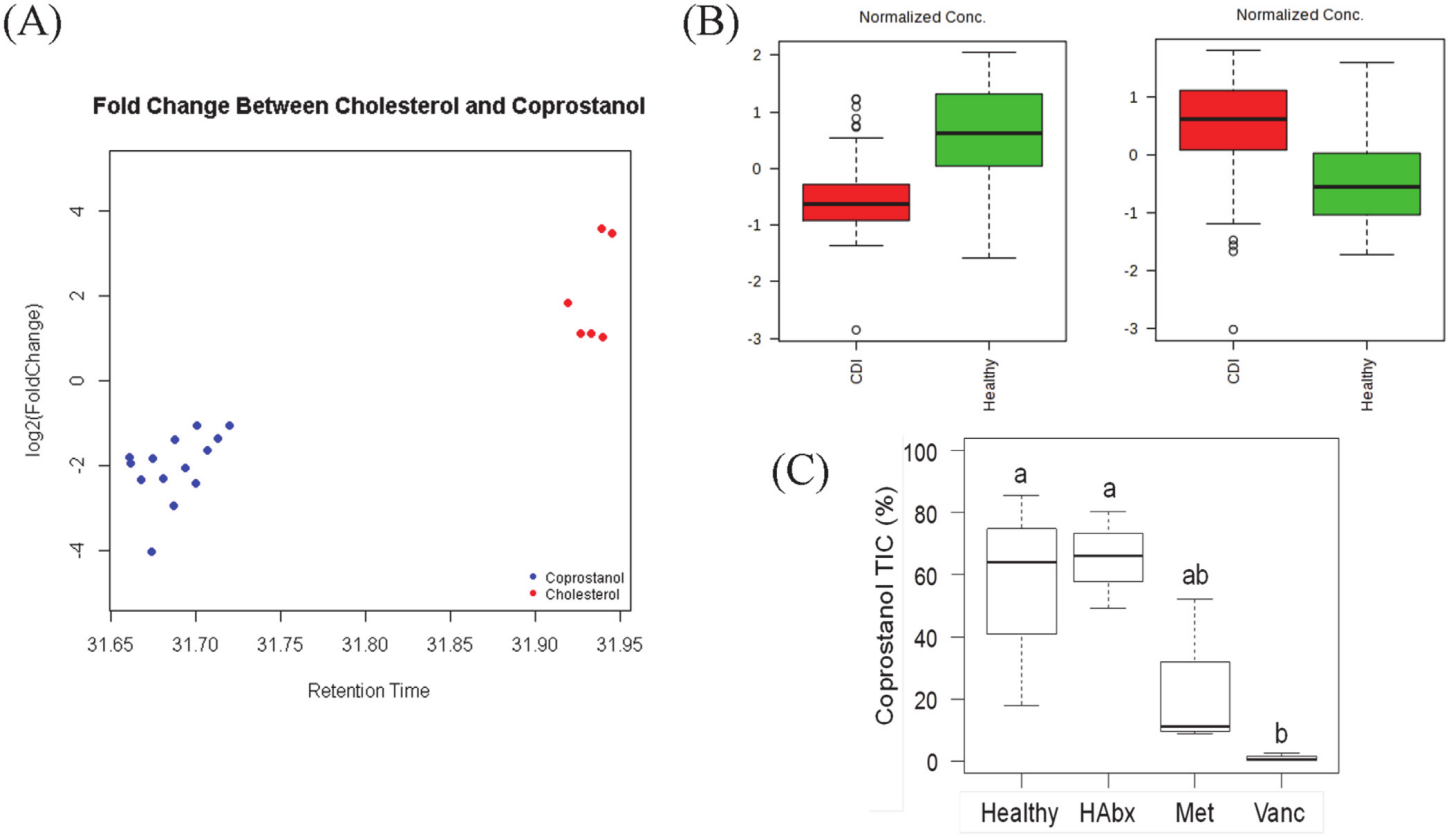
Inverse relationship of cholesterol and coprostanol levels in fecal extracts from subjects with CDI and Healthy controls. **(A)** The relationship between retention times for cholesterol and coprostanol as determined by mass spectrometry (x-axis) and their relative abundance (y-axis). The inverse relationship between the two compounds based on fold change in fecal composition is highlighted in blue and red circles. **(B)** Box-plots showing distribution of average total ion current of coprostanol (left) and cholesterol (right) for all fecal samples from the Healthy or the CDI group. The TIC of the two metabolites was normalized by auto-scaling before plotting. **(C)** Percentage of coprostanol TIC relative to the sum of coprostanol and cholesterol TIC for each subgroup. ANOVA on the ranked Coprostanol TIC values indicated a significant difference among the four cohorts (*F*_3, 9_ = 9.797, *p* < 0.01). For the 13 subjects, ranks were highest for Healthy (10) and HAbx (10), followed by Met (6) and Vanc (2); numbers in parentheses indicate mean ranks. Letters above whiskers indicate similar groups based on ranks according to the Tukey HSD test. Fecal samples from a Healthy, HAbx, or Metronidazole origin could be grouped together according to coprostanol levels. Likewise, Metronidazole and Vancomycin treated fecal derived samples could be grouped together based on coprostanol levels.

Using positive mode mass spectrometry, we identified the top two retention times as cholesterol (leading molecular ion [M^+^] of 386.4) and coprostanol ([M^+^] of 388.4), both of which had a >97% match to the NIST database (S1 Fig). Thus, we chose cholesterol and coprostanol as two metabolites that we could confidently characterize that distinguish CDI from Healthy fecal samples. The levels of these two compounds were inversely correlated with each other, and a subset of chromatograms derived from vancomycin treated CDI subjects showed high levels of cholesterol and very low levels of coprostanol (Fig 3A).

To compare proportional abundance of coprostanol and cholesterol between samples, we quantified peak areas in total ion current (TIC) for both metabolites in injected fecal extract. Grouped analysis using all samples revealed that coprostanol levels were highest and cholesterol levels were lowest in healthy subjects. In contrast, mean coprostanol level was lowest and cholesterol level was highest in subjects with CDI (p<0.001; Student’s *t* test) (Fig 3B). Subgroup analysis showed a significant difference in mean coprostanol total ion current among the four groups (F_3, 9_ = 9.797, p < 0.01, ANOVA). Healthy subjects (HAbx and Healthy) had significantly higher mean coprostanol TIC percentage than subjects with CDI (Metronidazole and Vancomycin subgroups) (Fig 3C). Additionally, HAbx, Healthy, and Metronidazole groups had mean coprostanol TIC percentage that were significantly greater than the Vancomycin group.

### NMDS (Non-metric multidimensional scaling) analysis identified 63 gut bacteria associated with coprostanol

**Fig 4:**
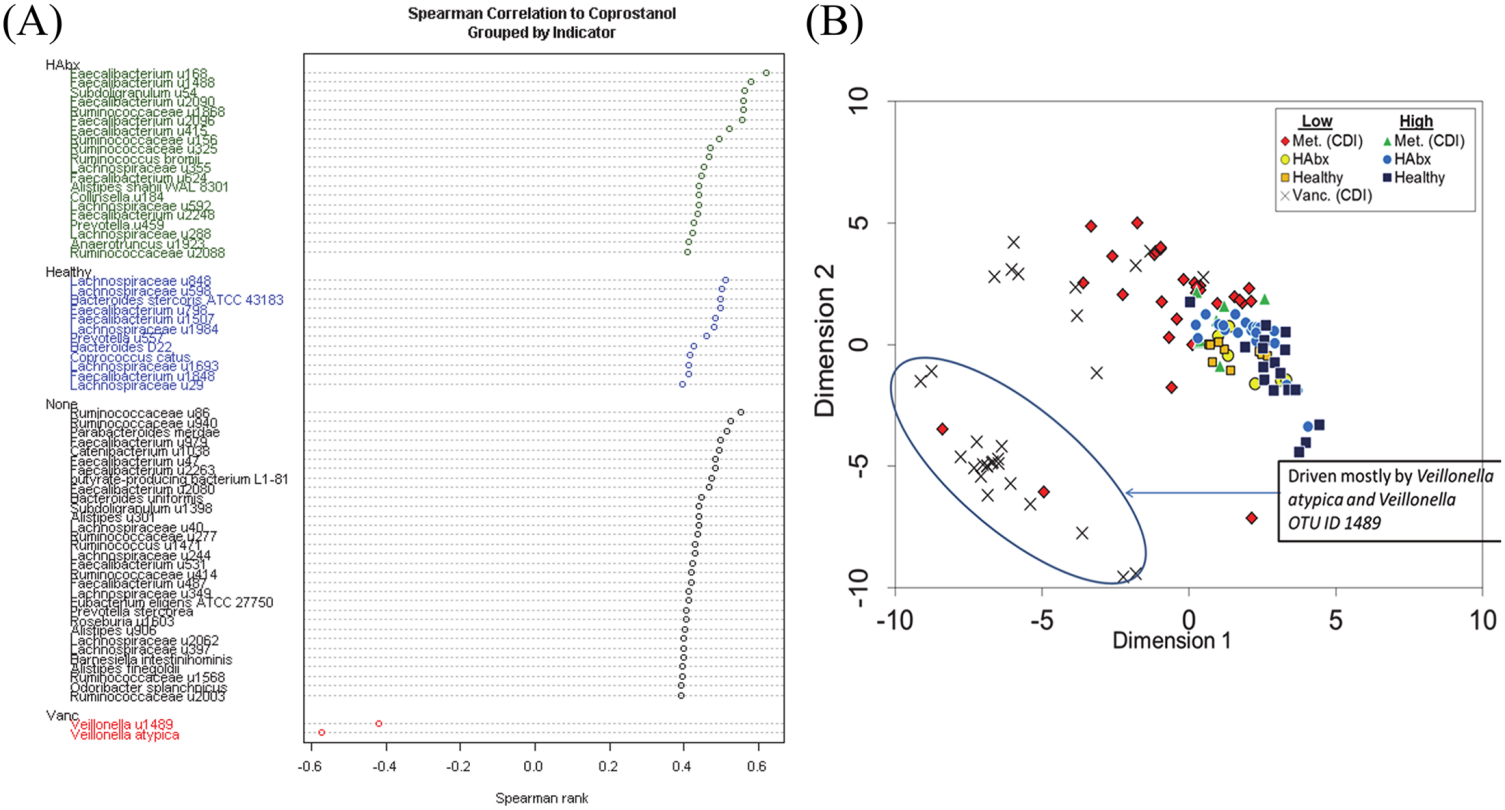
Correlation between coprostanol total ion current (TIC) and 16S rRNA taxonomic sequences. **(A)**: Spearman’s rank of 65 bacteria significantly correlated to coprostanol and cholesterol total ion current. Each taxon was grouped according to an indicator cohort (HAbx, Healthy, or Vancomycin) using indicator species analysis. No phylotypes were identified as an indicator for the Metronidazole (Met) cohort. **(B)** Nonmetric Multidimensional Scaling (NMDS) analysis of bacterial OTUs and relative coprostanol TICs. Fecal samples were assigned as either “High” or “Low” coprostanol formers. Data was reduced by the NMDS approach using Bray-Curtis distances, followed by Spearman rank correlation to identify OTUs associated with coprostanol TIC levels. Dimension 1 represents coprostanol levels; Dimension 2 represents CDI treatment or antibiotics exposure for each subject.

After bioinformatic classification of 16S reads was performed, a matrix of 2395 OTUs was reduced to two dimensions using a Bray-Curtis distance, which does not count zero values in the matrix as a sign of similarity. The method reduced the matrix of 2395 OTU’s into two dimensions (Stress = 0.165) and correlated at 0.747 with the original data matrix. This new 2-dimensional matrix was then regressed to High or Low coprostanol levels for each subject/time point based on TIC levels (see Methods section). After controlling for disease/drug status and variability associated with the repeated measures (time within subject), 66.5% of the remaining SSE (sum of square errors) could be attributed to coprostanol on NMDS axis 1 (F_2,103_ = 102.41, p <0.001). Coprostanol was not significantly related to the second axis (F_2,103_ = 0.572, p = 0.566), and explained only 1.1% of the variability after controlling for covariates. (Fig 4). In total, 63 bacterial OTUs correlated positively with coprostanol levels and two negatively correlated with coprostanol (Fig 4A, Table 2, and S3 Fig).

**Table 2:** Bacterial operational taxonomic units (OTUs) associated with coprostanol total ion current. For each OTU, the taxonomy (u = uncultured), Spearman correlation coefficient (*r*_s_), relative abundance (RA) and relative frequency (RF) based on 16S rRNA sequence reads are shown. A reference OTU (refOTU) sequence accession number from the Silva (release 108) database is shown for all uncultured species. Superscripts after the OTU number indicate whether the species is an indicator species for a specific cohort. HAbx: healthy subjects with prior antibiotic exposure, H: healthy subjects with no prior antibiotic exposure; Vanc: subjects with CDI who received vancomycin therapy. P-values are shown with Bonferroni correction.

Lowest Taxonomic Unit	*r*_*s*_	RA	RF	refOTU*	p-value**
*Veillonella atypica* ^**VANC**^	-0.573	0.112	0.345		7.991E-08
Veillonella u1489^**VANC**^	-0.418	0.02	0.159	JF226995	9.997E-03
Ruminococcaceae u2003	0.392	0.004	0.257	EU766380	4.126E-02
*Odoribacter splanchnicus*	0.392	0.006	0.363		4.119E-02
Ruminococcaceae u1568	0.394	0.006	0.159	FJ367680	3.753E-02
Lachnospiraceae u29^**H**^	0.395	0.005	0.274	DQ905864	3.599E-02
*Alistipes finegoldii*	0.396	0.007	0.389		3.451E-02
*Barnesiella intestinihominis*	0.397	0.009	0.265		3.201E-02
Lachnospiraceae u397	0.399	0.004	0.168	GQ897082	2.820E-02
Blautia u2062	0.399	0.004	0.363	FJ366849	2.820E-02
Alistipes u906	0.403	0.001	0.186	DQ805782	2.330E-02
Roseburia u1603	0.404	0.003	0.283	FJ363547	2.165E-02
*Prevotella stercorea*	0.407	0.009	0.168		1.793E-02
Ruminococcaceae u2088^**HAbx**^	0.409	0.003	0.133	JF052873	1.634E-02
*Eubacterium eligens* ATCC 27750	0.411	0.033	0.46		1.477E-02
Anaerotruncus u1923^**HAbx**^	0.412	0.003	0.133	FJ364566	1.389E-02
Faecalibacterium u1848^**H**^	0.412	0.005	0.106	DQ797991	1.360E-02
Lachnospiraceae u1693^**H**^	0.414	0.021	0.363	FJ512247	1.266E-02
Lachnospiraceae u349	0.414	0.006	0.265	GQ898687	1.261E-02
*Coprococcus catus*^**H**^	0.416	0.001	0.168		1.097E-02
Faecalibacterium u487	0.419	0.002	0.283	GQ898622	9.337E-03
Ruminococcaceae u414	0.419	0.002	0.133	EF403999	9.054E-03
Faecalibacterium u531	0.422	0.023	0.301	FJ682262	7.921E-03
Lachnospiraceae u288^**HAbx**^	0.424	0.004	0.168	EF404854	6.803E-03
Bacteroides D22H	0.425	0.007	0.531		5.635E-07
Prevotella u459^**HAbx**^	0.427	0.01	0.195	DQ904655	5.721E-03
Lachnospiraceae u244	0.429	0.003	0.159	EF403824	5.036E-03
Ruminococcus u1471	0.43	0.004	0.15	DQ807522	4.820E-03
Ruminococcaceae u277	0.436	0.009	0.283	EF401722	3.234E-03
Faecalibacterium u2248^**HAbx**^	0.437	0.005	0.168	FJ372335	3.062E-03
Lachnospiraceae u592^**HAbx**^	0.44	0.013	0.425	FJ685394	2.630E-03
Lachnospiraceae u40	0.44	0.01	0.159	DQ905337	2.602E-03
Alistipes u301	0.44	0.014	0.451	EF402337	2.593E-03
Collinsella u184^**HAbx**^	0.441	0.019	0.407	BAAZ01000335	2.504E-03
Subdoligranulum u1398	0.441	0.002	0.186	DQ802754	2.404E-03
Alistipes shahii WAL 8301^**HAbx**^	0.441	0.006	0.442		2.372E-03
Bacteroides uniformis	0.445	0.057	0.611		1.901E-03
Faecalibacterium u624^**HAbx**^	0.446	0.005	0.389	FJ684971	1.784E-03
Lachnospiraceae u355^**HAbx**^	0.454	0.001	0.133	EF400772	1.044E-03
Prevotella u557^**H**^	0.461	0.091	0.292	FJ685510	6.439E-04
*Ruminococcus bromii*^**HAbx**^	0.466	0.002	0.168		4.829E-04
Faecalibacterium u2080	0.466	0.004	0.363	FJ509845	4.651E-04
Ruminococcaceae u325^**HAbx**^	0.471	0.019	0.451	GQ897539	3.367E-04
butyrate-producing bacterium L1-81	0.475	0.012	0.345		2.611E-04
Lachnospiraceae u1984^**H**^	0.48	0.004	0.221	GQ491343	1.798E-04
Faecalibacterium u2263	0.484	0.007	0.442	JF220917	1.360E-04
Faecalibacterium u1507^**H**^	0.484	0.007	0.336	DQ806026	1.305E-04
Faecalibacterium u47	0.485	0.012	0.398	BAAU01000008	1.265E-04
Catenibacterium u1038	0.494	0.005	0.265	DQ809643	6.273E-05
Ruminococcaceae u156^**Habx**^	0.494	0.01	0.416	BAAZ01000004	6.224E-05
Faecalibacterium u979	0.496	0.018	0.372	DQ808727	5.661E-05
Faecalibacterium u798^**H**^	0.497	0.015	0.389	FJ675885	5.153E-05
Bacteroides stercoris ATCC 43183^**H**^	0.499	0.112	0.558		4.471E-05
Lachnospiraceae u598^**H**^	0.501	0.015	0.301	FJ679676	3.694E-05
Lachnospiraceae u848^**H**^	0.511	0.006	0.265	DQ796443	1.699E-05
Parabacteroides merdae	0.513	0.02	0.54		1.531E-05
Faecalibacterium u415^**Habx**^	0.52	0.014	0.442	AF499902	8.722E-06
Ruminococcaceae u940	0.525	0.023	0.363	FJ370833	5.610E-06
Ruminococcaceae u86	0.552	0.006	0.363	AB506402	5.635E-07
Faecalibacterium u2096^**Habx**^	0.555	0.009	0.389	FJ366675	4.089E-07
Ruminococcaceae u1868^**Habx**^	0.559	0.017	0.221	FJ369586	3.081E-07
Faecalibacterium u2090^**Habx**^	0.559	0.026	0.46	FJ511314	2.836E-07
Subdoligranulum u54^**Habx**^	0.562	0.008	0.319	DQ905758	2.193E-07
Faecalibacterium u1488^**Habx**^	0.578	0.014	0.442	DQ794141	4.917E-08
Faecalibacterium u168^**Habx**^	0.621	0.065	0.522	BAAV01000571	5.190E-10

* Sequence Accession number from the Silva database

** p-value from Spearman rank

**Fig 5:**
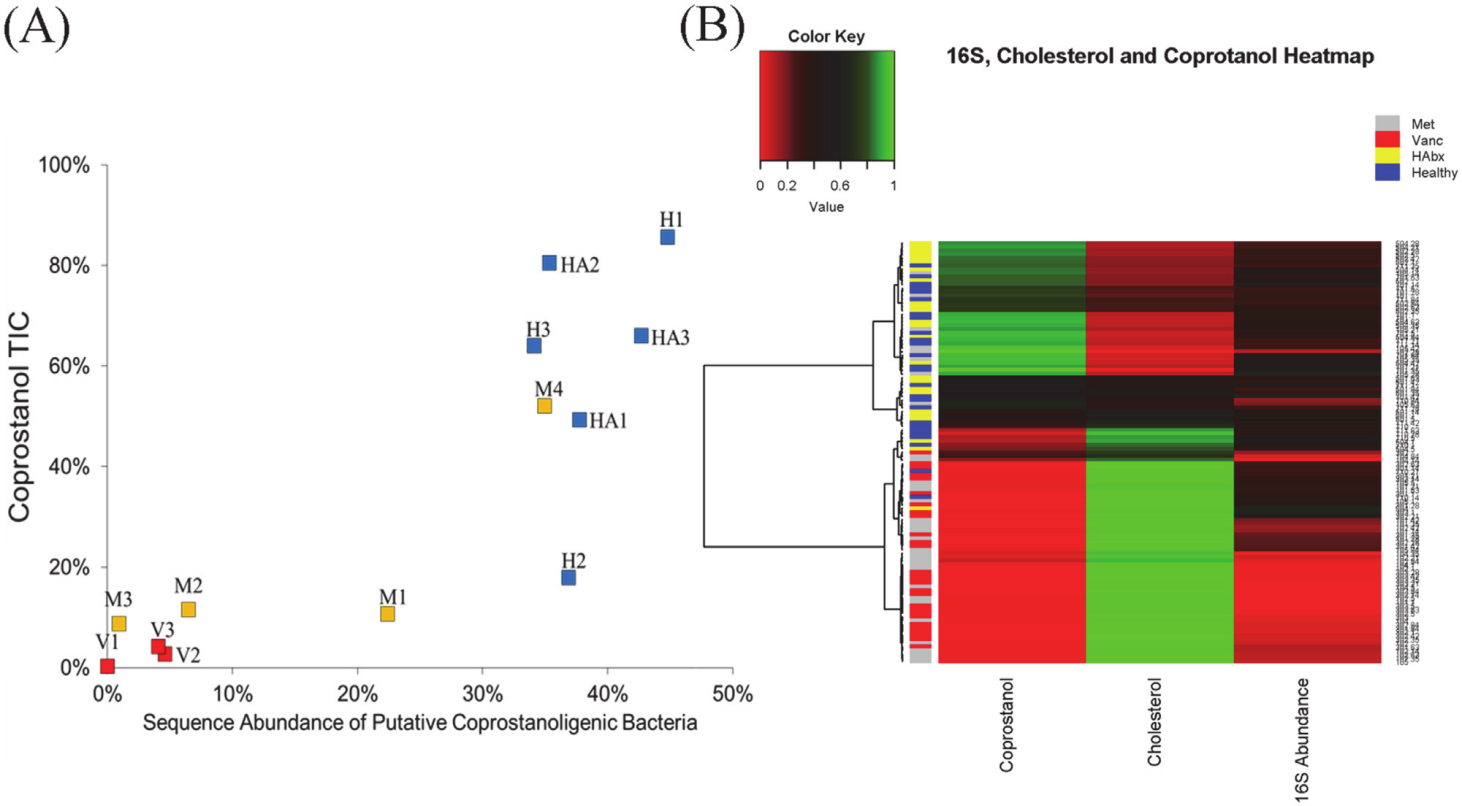
Relationship between the abundance of coprostanol-associated bacteria and coprostanol levels for each subject. **(A)** The mean abundance of coprostanol-associated bacteria for all samples within each subject is shown in x-axis. The mean proportion of coprostanol relative to the sum of cholesterol and coprostanol in TIC (total ion current) is shown on the y-axis. Both an exponential and a linear model fit well to the correlation between coprostanol TIC and 16S rRNA sequence abundance (see Results section). H: Healthy volunteers without prior antibiotic use, HA: Healthy volunteers with prior antibiotic exposure, M: Subjects with CDI who received metronidazole therapy, and V: subjects with CDI who received oral vancomycin therapy (V). **(B)**: Hierarchical clustering of all samples using Ward’s clustering on a Manhattan distance matrix. Each row indicates a sample, color-coded according to cohort: HAbx (yellow), Healthy (blue), Metronidazole group (gray), and Vancomycin group (red). The abundance of coprostanol, cholesterol TIC, and 16S rRNA sequence reads that mapped to coprostanol-associated bacteria for each sample is shown per each column.

The NMDS analysis revealed two predominant clusters. One cluster was dominated by the Vancomycin cohort and was driven primarily by two *Veillonella* species that correlated negatively with coprostanol (Fig 4B). We examined the relationship between the levels of coprostanol (TIC’s) and the relative abundance of coprostanol-associated gut bacteria (i.e. total abundance of 63 coprostanol-associated bacteria based on 16S rRNA sequence reads). A strong, positive correlation between levels of fecal coprostanol and the abundance of coprostanol-associated bacteria in healthy subjects could be modeled monotonically (*r*_s_ = 0.868, *p*<0.001) (Fig 5). Both a linear (*R*^2^ = 0.731) and an exponential (*R*^2^ = 0.736) model fit equally well to the correlation between these two metrics, although inter-individual and longitudinal variations between coprostanol, cholesterol levels, and abundance of coprostanol-associated bacteria were also observed (S4–S16 Figs). With the exception of one subject (M4) in the metronidazole group, the abundance of coprostanol-associated bacteria and coprostanol levels in CDI subjects were generally low compared to healthy controls. The depletion of coprostanol-associated bacteria and low coprostanol levels were particularly pronounced in the vancomycin subgroup (Fig 5).

### The association of specific gut bacteria with individual cohorts

We asked which of the 65 bacterial OTUs identified by the NMDS analysis were associated with each of the four subject cohorts. Using Indicator Species Analysis (ISA), we found that 31 of 65 bacterial OTUs were associated with health. Of these, 20 phylotypes were “indicators” of healthy subjects with antibiotic exposure, and 11 were associated with healthy subjects without prior antibiotic exposure, respectively. Two OTUs were associated with CDI subjects who received vancomycin. No indicator species were found for the metronidazole subgroup (Fig 4A, Table 2). In total, 33 OTUs were associated with one of the four cohorts (S3 Fig). Next, we combined the vancomycin and metronidazole subgroups as the “disease” group and the Healthy and HAbx subgroups as the “health” group, and asked whether the remaining 32 OTUs could be indicators of health or disease. Of the 32 OTUs, 19 were indicators of “health” (Table 3), but no additional indicator species were identified for the “disease” group. The remaining 12 OTUs that were associated with coprostanol could not be assigned as an indicator for any of the four subject cohorts.

**Table 3:** Indicator species analysis (ISA) of the remaining 32 OTUs that were not previously assigned to one of the four individual cohorts. Groups were designated as “Health” for healthy volunteers (combining the two groups with and without prior antibiotic exposure, and as “Disease” for subjects with CDI (combining the metronidazole and vancomycin groups). A representative sequence accession number from Silva (release 108) database is shown for all uncultured species as a reference OTU. Indicator value of species *j* in group *k* is the product of the percent relative abundance of each organism to a specific cohort along with its percent relative frequency. Those species that are significant after 100,000 Monte-Carlo randomizations of ecological communities are listed below.

			Indicator Value (%)		Relative Abundance (%)		Relative Frequency (%)	
Max Group	Lowest Taxonomic Unit*	refOTU**	No Disease	Disease	No Disease	Disease	No Disease	Disease
No Disease	Faecalibacterium u2263	JF220917	75.7	0.6	96.5	3.5	78.4	16.1
	Faecalibacterium u47	BAAU01000008	75.3	0.1	98.5	1.5	76.5	9.7
	*Eubacterium eligens* ATCC 27750		66.7	2.8	75.6	24.4	88.2	11.3
	Catenibacterium u1038	DQ809643	55.1	0.1	96.9	3.1	56.9	1.6
	butyrate producing bacterium L1 81		54.9	1	93.3	6.7	58.8	14.5
	Faecalibacterium u2080	FJ509845	54.8	1.9	79.9	20.1	68.6	9.7
	Faecalibacterium u979	DQ808727	49.7	3.1	72.4	27.6	68.6	11.3
	Faecalibacterium u487	GQ898622	45.5	1	89.2	10.8	51	9.7
	Ruminococcaceae u86	AB506402	38.9	6.1	70.9	29.1	54.9	21
	Subdoligranulum u1398	DQ802754	38	0	97	3	39.2	1.6
	Lachnospiraceae u397	GQ897082	37.3	0	100	0	37.3	0
	Roseburia u1603	FJ363547	35.6	3.1	78.8	21.2	45.1	14.5
	*Prevotella stercorea*		35.1	0	99.6	0.4	35.3	1.6
	Lachnospiraceae u.349	GQ898687	34.4	2.4	70.1	29.9	49	8.1
	Lachnospiraceae u. 244	EF403824	33.3	0	99.8	0.2	33.3	1.6
	Ruminococcus u.1471	DQ807522	30.8	0	98.3	1.7	31.4	1.6
	Ruminococcaceae u.414	EF403999	29.4	0	100	0	29.4	0
	Lachnospiraceae u.40	DQ905337	27.2	0.3	81.5	18.5	33.3	1.6
	Alistipe*s* u.906	DQ805782	27.0	1.1	86.2	13.8	31.4	8.1
Disease	None		--	--	--	--	--	--
None	*Bacteroides uniformis*		37.0	23.5	43.9	56.1	84.3	41.9
	*Alistipes finegoldii*		33.8	9.8	59.4	40.6	56.9	24.2
	*Parabacteroides merdae*		33.2	21	45.8	54.2	72.5	38.7
	Blautia u2062	FJ366849	29.9	8.2	49.2	50.8	60.8	16.1
	Alistipes u301	EF402337	28.8	15	42	58	68.6	25.8
	Ruminococcaceae u2003	EU766380	25.6	4.5	76.8	23.2	33.3	19.4
	Ruminococcaceae u940	FJ370833	24.6	12.8	50.3	49.7	49	25.8
	*Barnesiella intestinihominis*		19.8	3.9	38.9	61.1	51	6.5
	Ruminococcaceae u277	EF401722	16.1	12.3	45.7	54.3	35.3	22.6
	*Odoribacter splanchnicus*		14.5	18.2	29.6	70.4	49	25.8
	Faecalibacterium u.531	FJ682262	9.2	9.3	17.4	82.6	52.9	11.3
	Ruminococcaceae u1568	FJ365212	9.1	6.5	42.4	57.6	21.6	11.3

* u = uncultured followed by OTU number from the Silva database

** Accession number based on Silva (release 108)

### Co-occurrence of coprostanol-associated bacteria in gut microbiota

**Fig 6:**
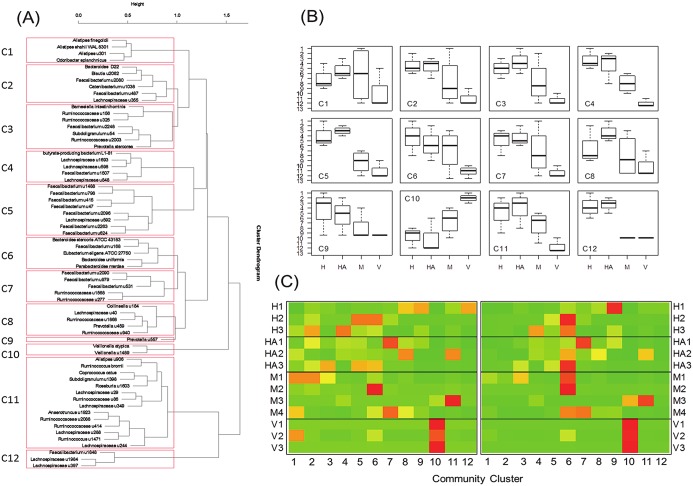
Agglomerative hierarchical clustering of 63 OTUs correlated with coprostanol and two correlated with high cholesterol. The dendrogram shows communities of bacteria more likely to co-localize with each other based on rank coprostanol and cholesterol GC-MS levels. Communities are shown in red boxes and are numbered from community 1 to 12 (C1-C12) (B) Boxplots for ranks of the abundances of community clusters (C1-C12). Highest abundances were given the lowest rankings (i.e., 1 = most abundant). The rank abundances were determined based on disease-drug combination (i.e., cohort). Cohort abbreviations are health (H, *n* = 3), healthy with prior antibiotics (HA, *n* = 3), CDI with metronidazole treatment (M, *n* = 4), and CDI with vancomycin treatment (V). Clusters are defined as in Fig 6A in red boxes. (C) Heatmaps for subject by community clusters scaled by column (left) and by row (right). Color scale goes from green (low value) to red (high value). Column scaling (left) indicates in which subject(s) a particular community cluster is dominant. Row scaling indicates which community clusters any given subject was dominated by, if any. Heatmap on the left indicates clustering based on the two-level model (CDI vs Health) and heatmap on the right is clustering based on a 4-level model (H vs. HA vs. M vs. V).

To identify bacterial communities that co-occur more frequently than they are in combination with other members of the 65-OTU community, we performed an agglomerative hierarchical clustering of the 65 coprostanol-associated bacteria. Using this algorithm, we identified 12 clusters of microbial communities ranging from a single species community to a 14-member community (Fig 6). The 12 community clusters determined from hierarchical clustering analysis was found in a majority of subjects studied, though the presence of all 12 communities was not accounted for in every subject. Furthermore, each hierarchically clustered community contained both our proposed coprostanoligenic as well as non-coprostanoligenic bacteria. We performed a nested ANOVA on ranked 16S rRNA sequence data after summing the sequences for each cluster to generate a total cluster score. In all cases, there was a significant cohort effect and significant difference of subjects within cohort, but not for time except for clusters 8 and 11. This provided confidence that we could collapse the nested variability by averaging a single cluster score for subjects based on their longitudinal data. This new collapsed dataset was then subjected to a separate ANOVA. ANOVA testing two separate models for each cluster (Healthy vs CDI), and the other with a single factor having four levels (Healthy, HAbx, Vanc, Met) on the ranked abundance for each cluster was performed. This analysis showed that clusters 3–5 and 10–12 could statistically distinguish for the two level model while 2–5, 7, and 9–12 could distinguish between the four level model (*p* < 0.05; Fig 6).

## Discussion

In healthy populations, coprostanol conversion in the gastrointestinal tract is influenced by demographics and gender [27–31]. Midtvedt et al. showed that coprostanol conversion phenotype is established early in the first year of life [32]. However, antibiotic treatment can influence the rate of conversion [33]. Healthy individuals can be classified as high or low coprostanol formers [34, 35], and these high or low metabolic phenotypes could be replicated in animals by transplantation of human fecal material into gnotobiotic rats [36]. These observations suggest that coprostanol-associated phenotype is determined by the composition of gut microbiota. However, the identity of gut microbes that can reduce cholesterol to coprostanol has not been well defined. By correlating 16S rRNA microbiome and GC-MS metabolomics datasets, we identified 65 gut bacteria associated with fecal coprostanol and cholesterol. Given the recent report that showed an association between specific intestinal microbiota and blood lipid levels in human subjects [37], and the discovery of a new cholesterol reducing species *Bacteroides sp*. D8 [38], cultivation and functional analysis of coprostanol-associated bacteria identified in our study would be of great interest in cardiovascular disease.

A majority of bacterial phylotypes associated with high coprostanol levels belonged to the *Lachnospiraceae* and *Ruminococcaceae* family of *Clostridiales* order, suggesting that some members of these families may harbor coprostanoligenic activity. Fu *et al*. recently analyzed the bacterial taxa of a large population cohort and identified 34 gut bacterial taxa that were strongly associated with blood lipid levels. These investigators showed that the gut microbiome explained a large percentage of variations in blood HDL levels independent of age, gender and host genetics. Interestingly, 30 of 34 bacteria taxa identified in this study belonged to the *Clostridiales* order, most of which were either *Lachnospiraceae* or *Ruminococcaceae* (Tables 2 and 3). Thus, our results are consistent with these findings pertaining to the association of health with many of these putative coprostanoligenic bacteria. It also portends to study the relationship, if any, between the levels of these gut microbes identified here and blood levels of cholesterol in a susceptible CDI population.

The majority of *Lachnospiraceae* and *Ruminococcaceae* species identified in Table 2 and Fig 4A have not been previously cultivated, making functional assays to confirm coprostanoligenic activities challenging. Nonetheless, since some of these organisms were more likely to co-occur in community clusters (Fig 6), they may share similar physiologic, functional or growth requirements. As an example, conditions that are favorable to *Barnesiella intestinihomis* and *Prevotella stercorea*, may be favorable to the other five co-occurring uncultured bacteria (Fig 6; See Group #3), since metabolism of a functional gut ecosystem depends on cross feeding of bacteria to generate metabolites. For instance, the nitrogen cycle requires communities of microbes where species act in succession whereby the metabolic product of one species feeds directly into the metabolic reactant of another [39].

Human gut microbiota is known to confer colonization resistance against *C*. *difficile*, but the underlying mechanisms remain poorly understood. Recent data suggest that resistance mechanisms may involve cholesterol and bile acids metabolisms [40]. Schwan et al. showed that entry of *C*. *difficile* to colonocytes is cholesterol dependent and can be inhibited by methyl-β-cyclodextrin that depletes cholesterol [41]. *Clostridium difficile* toxin A binding to target cells is facilitated by cholesterol-enriched lipid rafts [42] and a number of sterols and bile acids can inhibit *C*. *difficile* binding [43,44]. Clinically, the use of statins (which inhibit cholesterol biosynthesis) is associated with a reduced risk of *Clostridium difficile* infection [45, 46]. While the precise mechanisms underlying colonization resistance remain unknown, low levels of luminal coprostanol and high levels of cholesterol in the setting of altered gut microbiota may play a role in the susceptibility to *C*. *difficile* infection.

The role for bile acids metabolism in *C*. *difficile* pathogenesis is also of significance. Bile acids are the main metabolites of cholesterol in the liver. Primary bile acids (e.g. cholic acid and chenodeoxycholic acid) are produced by endogenous enzymes in the liver and conjugated to taurine or glycine to form bile salts to assist in lipid digestion in the small intestine. About 5% of the secreted bile salts reach the colon (95% are reabsorbed via the enterohepatic circulation), where primary bile acids are deconjugated and dehydroxylated by intestinal microbiota to form secondary bile acids deoxycholic and lithocholic acids. Therefore, antibiotics that alter gut microbiota are expected to reduce the transformation of primary bile acids into secondary bile acids [47]. In our study, the depletion of these essential commensals may have led to elevated fecal cholesterol especially in the CDI cohort administered vancomycin. Since vancomycin treatment is generally considered for more severe form of infection, we speculate the loss of these commensals leads to the loss of bile acid components that ultimately gave rise to abnormal fecal cholesterol levels seen here.

Interestingly, primary bile acids are potent germinates for *C*. *difficile* spores to transform into vegetative bacteria [48]. In contrast, secondary bile salts, which are generated by gut microbes through enzymatic action of primary bile acids, inhibit the vegetative growth of *C*. *difficile in vitro* [49], and likely also inhibit germination and/or vegetative growth *in vivo*. In patients treated with fecal microbiota transplant for recurrent CDI, an increase in deoxycholic acid and lithocholic acid was observed, which was accompanied by an increase in *Clostridial* clusters IV and XIVa (which include members of the *Lachnospiraceae* and *Ruminococcaceae* families). Members of these families harbor numerous genes for 7α-dehydroxylation and deconjugation of primary bile acids [40]. Administration of *Clostridium scindens*, an organism expressing enzymes involved in secondary bile acid generation, enhance resistance to *C*. *difficile* infection in mice [50, 51]. Taken together, perturbation of gut microbiota may decrease the conversion of primary bile salts to secondary bile salts, leading to increased vegetative growth, toxin production and colitis. Consistent with this, we have previously shown that members of the *Lachnospiraceae* and *Ruminococcaceae* families are depleted in patients with CDI [2]. In the present study, many OTUs that belonged to these two families correlated strongly with high coprostanol levels and were indicator species for health (Fig 4A).

Spearman correlation and NMDS analysis revealed a negative correlation between coprostanol level and two *Veillonella* species (Figs 4 and 5). Endogenous to the oral cavity, *Veillonella spp*. have also been found in atherosclerotic plaques, fecal samples, and oral washings from subjects with known cardiac events [52]. These observations suggest an association between *Veillonella* and cholesterol, and are consistent with our data indicating a reduction of coprostanol levels that was selectively associated within the vancomycin subgroup (see S7–S9 Figs). Some bacteria may travel to target sites in the body by cholesterol-laden foam cells [53], suggesting that cholesterol may be exploited for additional functionality by pathogenic gut microbes. It should be noted that variance exists between subject cohorts and time points (see S4–S16 Figs) and wide dispersion exists amongst Spearman correlations of specific individuals compared to Spearman correlations within a specific cohort. Despite this, a global assessment of metabolite and microbial features shows healthy subjects having more candidate bacteria associated with high fecal coprostanol levels than CDI subjects do. Separate evidence in our lab based on sequencing a larger cohort of CDI subjects longitudinally have shown local fluctuations within day-to-day sampling, but global stability over 100 days (manuscript in progress). This dynamical trend of local instability and global equilibrium was also found in a recent analysis of CDI microbiota recovery after fecal microbial transplantation (FMT) [54]

In summary, we have identified 63 human endogenous gut microbes associated with coprostanol. While our study points towards an association of the human microbiome towards cholesterol metabolism, further studies should focus on assessing roles of these candidate bacteria on *C*.*difficle* recovery in animal studies and a function of fecal coprostanol/cholesterol ratio in such a recovery. Modalities involving shotgun or whole genome sequencing on fecal extracts to identify differential genes involved in cholesterol metabolism between CDI and healthy controls can extend our findings to cholesterol and bile-acid pathways absent in CDI due to antibiotic perturbation. In short, microbiota-mediated transformation of fecal cholesterol to coprostanol may enhance resistance to *C*. *difficile* infection by altering toxin entry and decreasing the availability of cholesterol substrates for primary bile acids generation. Given the recent report that suggests a role for gut microbiome in blood lipid levels [37] and the inverse relationship between serum cholesterol levels and fecal coprostanol/cholesterol ratio [22], cultivation and functional analysis of coprostanol–associated bacteria identified in our study would be of great interest for both cardiovascular disease and *C*. *difficile* infection.

## Materials and Methods

### Subject recruitment and longitudinal fecal sampling

Fecal samples (mean of 8.7 fecal samples per subject) were collected longitudinally from 13 subjects over a three-month period. Time between sample collections ranged from nine to 17 days with a mean of 11 days. Fecal samples were collected for a period up to 90 days from years 2011–2013. Subject characteristics and prior antibiotic history for all 13 participants are shown in Table 1. Of the 7 subjects, 3 subjects were treated with vancomycin (Vanc) and 4 subjects received metronidazole therapy (Met) for their underlying CDI pathology. Healthy subjects were recruited and analyzed according to prior history of antibiotic exposure (within three months of the first fecal sample collection): three subjects had prior antibiotic exposure (HAbx), and three subjects had no prior antibiotic exposure (Healthy). All subjects were recruited from University of Florida Health System in Gainesville, FL as part of a larger longitudinal *Clostridium difficile* gut microbiome study. The University of Florida IRB approved the study, and all subjects provided written informed consent to participate.

### 16S rRNA Sequencing and Bioinformatics analysis

Fecal samples delivered to the laboratory via postal service or through the study coordinator were immediately aliquoted in 1.5mL Eppendorf tubes, assigned a code for a de-identification, and stored at -80°C until genomic DNA extraction was to be performed. Fecal samples were contained in Invitek PSP Stool Collection vials that held a capacity of 10mL of fecal material with stabilization buffer. Genomic DNA extraction using a bead beating technique, bacterial 16S rRNA gene amplification, and sequence analysis were performed as described previously (2). Briefly, the V1-V3 hypervariable 16S rRNA gene was amplified in quadruplicate using barcoded PCR primers with denaturing and amplification times derived from the Human Microbiome Project (www.hmpdacc.org). The primers contained a titanium-flex region for compatibility to the Roche/FLX sequencer. Amplicons were excised under fluorescence, gel purified, (Qiagen gel extraction kit), quantified using Qubit (Invitrogen), and pooled in equimolar concentrations of 100ng per amplicon. Sequencing was performed on the Roche/454 FLX pyrosequencing platform. Using a custom bioinformatic pipeline [2], 2,395 unique reference operational taxonomic units (refOTUs) were obtained post-filtering based on 97% sequence similarity to the Silva 16S rRNA database. The taxonomy at the nearest phylogenetic level for all 2,395 16s RNA obtained in this study are shown in S1 Table.

### GC-MS Sample Preparation

A dichloromethane microextraction was performed on 1 mL aliquot of each fecal sample from the PSP^®^ kit (Invitek, Germany) that was used for 16 rRNA deep sequencing. Each aliquot was vortexed and placed in a 13x15 mm glass test tube with screw-top lid and 1 mL (1:1 vol/vol) of dichloromethane was added using a gas-tight Hamilton syringe and vortexed for 1 minute. Samples were then centrifuged at ~3000g for 20 min at 4°C, and the organic fraction was removed using a glass pipette. The resultant organic layer was then transferred to GC-MS auto sampler vials and clamped with a PTFE faced rubber cap. The volume obtained from each extraction was variable; for small volumes, samples were placed in vial inserts. The extracted organic phase was diluted 1:100 and run on an Agilent 5973 GC-MS with auto sampler. 1μL of sample was injected (splitless) onto an HP-5MS capillary column (0.25mm x 30mm x 25μm) with helium as the carrier gas flowing at 1 mL/min. The solvent delay time was 6 minutes. An oven temperature was set at 30°C for 3 minutes and ramped to 320°C at 10°C/min until finally held for 10 minutes. Raw GC-MS extension files acquired from the instrument were converted to compatible files using Xcalibur v8 (ThermoScientific) and subsequently manipulated and processed in OpenChrom (San Diego, CA). A representative GC-fecal chromatogram, mass spectrometry fragmentation of both cholesterol and coprostanol, and molecular structures of the two sterols are shown in S1 Fig.

### Metabolomic Analysis

In order to extract differentially abundant features, peak areas for all chromatograms were combined into a format for discriminant analysis using the Metaboanalyst software suite [25, 26]. A matrix containing all 118 samples as row header and retention times as column header was generated by merging all raw data files. Each sample and sample time point was annotated as either CDI or Healthy (for a 2-component model), or Healthy, HAbx, Met, Vanc (for a 4-component model). The elements for this array input were peak intensities in units of total ion current (TIC). Using the Metaboanalyst suite, each spectrum was normalized by sum filtering and autoscaled, which resulted in a Gaussian distribution of intensities for all chromatograms. The output determined retention times that were significantly different between CDI and healthy cohort (irrespective of prior antibiotic exposure and antibiotic treatment) and VIP scores of retention times that contributed to the PLS-DA. 155 retention times were found to be statistically significant (*t* test). From the *t* test and VIP scores, retention times 31.700± 0.1000 mins and 31.900 ± 0.1000 mins were the top features that distinguished both the two and the four-component model. The chemical identity of these two peaks was investigated by mass spectrometry (Chemstation, Agilent), and compounds were matched to cholesterol and coprostanol for all subjects manually to avoid complications from retention times overlapping (see Results section). Each chromatogram was then deconvoluted by extracting the leading molecular ions for both molecules: cholesterol (*m/z* 353.4, 368.4, 386.4), and coprostanol (*m/z* 355.4, 373.4, 388.4) using XCalibur v8. The percentage of coprostanol as a function of the sum of coprostanol and cholesterol TIC was then computed for each chromatogram. A Spearman’s rank (rho coefficient) was calculated for each sample from all time points (S4–S16 Figs)

Other discriminating metabolites identified in this targeted screen that were identified included Vitamin E (RT 32.10 ± 0.150 mins), fatty acids hexadecanoic acid and octadecanoic acid (22.00–24.00 mins), and squalene ~33.00–34.00 mins. In addition, we observed that cholestanone, another cholesterol derivative, co-eluted with coprostanol peaks in some but not all chromatograms. However, due to high inter-individual variation of these other metabolites, the lack of agreement and ambiguity with NIST database match, and their relatively low overall contribution to PLS-DA VIP components, we selected cholesterol and coprostanol for subsequent analysis, as identification of these two metabolites was consistent across all 118 fecal samples.

### Nonmetric Multidimensional Scaling (NMDS)

To identify bacterial OTUs associated with cholesterol and coprostanol levels, two complementary methods were employed. First, Nonmetric Multidimensional Scaling (NMDS) using a Bray-Curtis distance was used as a data reduction technique. The original community matrix of 118 individual-time samples by 2395 bacterial OTUs, was reduced to two orthogonal dimensions using NMDS. The NMDS algorithm generated a best solution after 1000 random starts of 2-D matrices into a 118 by 2 two-dimensional matrix (Stress = 0.165). This allowed for a statistical analysis using ANOVA to identify unique bacterial species associated with one of the two dimensions. As an additional step, a Spearman rank correlation test determined which of the 2395 OTU's were most strongly related to the NMDS dimensions in order to indicate which OTU’s were related to coprostanol TIC levels. Spearman rank correlation, rather than a parametric alternative, was used because (a) there was a large number of zeroes in the community matrix, and (b) we had no *a priori* reason to suspect a linear relationship between OTUs and coprostanol or cholesterol levels. Since most samples had some amounts of cholesterol and coprostanol in their chromatograms, we designated “High” coprostanol formers as those whose ratio of coprostanol/(coprostanol + cholesterol) TIC was >50%, and “Low” coprostanol formers as those whose ratio was <50%. Each sample was then graphed in a NMDS plot as either a “High” or a “Low” coprostanol former.

### Indicator Species Analysis (ISA)

Indicator species analysis (ISA) [55] was used to identify bacterial OTUs (from NMDS and Spearman rank) that were significantly associated with a specific cohort. The procedure is a statistical method for determining whether a particular species is found in predefined groups significantly more likely than if the same species were randomly assigned to the groups. An ISA combines measures of relative abundance (i.e., the number of species in one group relative to that species abundance in all groups) and relative frequency (i.e., the proportion of samples the species occurs at least once in a group relative to the proportion of samples the species occurs in all groups) into a single measure (i.e., an indicator value). The indicator value is computed as *IV*_*kj*_ *= 100(RA*_*kj*_ *x RF*_*kj*_*)* where the *IV*_*kj*_ is the indicator value associated with each species *j* in each group *k*, *RA*_*kj*_ the relative abundance of each species *j* in each group *k* relative to all other groups, and *RF*_*kj*_ is the relative frequency of species *j* in each sample of group *k* relative to all other groups. Indicator values range from 0 (no indication) to 100 (perfect indication). Once indicator values were assigned for each species to each group, a Monte Carlo randomization procedure determined whether observed indicator values are higher than indicator values associated with randomized ecological communities [55]. We used 100,000 randomizations and determined our *IV*_*kj*_ significant if it gave a higher *IV*_*kj*_ than 95% of the randomizations after a Bonferroni correction for experiment-wise error. Probability values were adjusted using Bonferroni correction. The R package *labdsv* was used for ISA and ran on R console v3.1.2.

ISA was performed on coprostanol-associated bacteria with three sequential strategies in defining groups. In the first strategy, we used cohort groupings (i.e. Met, Vanc, HAbx, and Healthy). In the second strategy, any non-significant coprostanol-associated bacteria that could not be placed into one of the 4 initial groups were used in a second ISA using disease (combining Met and Vanc subgroups) or no disease (combining HAbx and Healthy subgroups) groupings. In the final ISA strategy, bacteria were analyzed without a priori defined groups (i.e., cohorts or disease), but groups were determined using the method of Dufrene and Legendre (1997) which relies on agglomerative clustering to determine independent community clusters. (see section on Agglomerative hierarchical clustering below)

### Agglomerative hierarchical clustering

We used agglomerative hierarchical clustering on a distance matrix generated from a species by sample matrix. The method finds groups that are most similar, combines them, and then combines the combined groups into higher clumps until all species are accounted for. The method produces a dendrogram (Fig 6) with nodes that divide species groups hierarchically into fewer and fewer different groups. The number of unique communities can be objectively determined when an ISA is run within each different number of possible groupings and the average *p*-value is returned for the entire sample. The method of Dufrene and Legendre [55] was used to combine agglomerative hierarchal clustering of taxa with ISA. The most parsimonious number of groups is the number of groups with the lowest average *p*-value resulting from the ISA. As recommended by [56], we used the flexible beta method with β = -0.25 on the Bray Curtis distance matrix. A Bray Curtis distance gives the proportion of shared taxa weighted by their abundance between any two samples. It does not count shared zeroes as a similarity and as such has applicable use with sparse matrices such as those generated by 16S rRNA sequencing.

## Supporting Information

S1 Fig GC-MS reveals cholesterol and coprostanol as differential metabolites in CDI. (A) Representative GC-chromatogram of typical healthy subject time point (above) and CDI subject time point (below). SF is the solvent front and FA represents fatty acid peaks that consistently eluted in the time window but whose identity and features could not be discriminated. (B) Weighted sum of partial least square regression coefficients for cholesterol and coprostanol with their inverse abundance for each of the four cohorts examined in this study (C) Chemical structure of cholesterol and coprostanol that were discriminating features between CDI and healthy longitudinal fecal samples. Right hand panel: Fragmentation pattern coprostanol and cholesterol with leading M+ ion (m/z) shown in red.
(PNG)

S2 Fig Identification of RTs (retention times) discriminating between CDI subjects taking Metronidazole, Vancomycin and Healthy controls. As an approach to identify compounds of CDI and Healthy, differential RTs were sought after. **(A)** PLS-DA VIP scores of the ten highest rank retention times with their relative abundances in healthy subjects with antibiotic history (HAbx), Healthy subjects with no reported antibiotic history (Healthy), and CDI subjects taking Metronidazole (Met) and Vancomycin (Vanc). **(B)** The contribution of the top VIP scores identified as cholesterol and coprostanol to the PLS-DA loadings according to antibiotic exposure and CDI-drug exposure (Healthy vs HAbx vs Met vs Vanc). The dispersion of retention times across the three principal components suggests some Healthy, HAbx, and Metronidazole fecal samples contained both cholesterol and coprostanol during the 90 days of longitudinal analysis.
(PNG)

S3 Fig Bacterial OTUs identified as indicator species for each cohort. Species related to either high coprostanol or low coprostanol were grouped by subject cohort (Healthy, HAbx, or Vanc). The indicator value (IV), relative abundance (RA), and relative frequency (RF) are shown in vertical axis and are sorted horizontally according to their indicator values. The Met cohort contained no indicator species. **Top:** 1 = *Lachnospiraceae u*. *OTU ID 1693*, *2* = *Prevotella u*. *OTU ID 557*, 3 = *Faecalibacterium u*. *OTU ID 798*, 4 = *Lachnospiraceae u*. *OTU ID 848*, 5 = *Lachnospiraceae u*. *OTU ID 1984*, 6 = *Faecalibacterium u*. *OTU ID 1507*, 7 = *Eubacterium eligens ATCC 27750*, 8 = *Bacteroides D22*, 9 = *Bacteroides stercoris ATCC*.*43183*, 10 = *Faecalibacterium u*. *OTU ID 1848*, 11 = *Coprococcus catus*. **Middle:** 1 = *Faecalibacterium* u. OTU ID 2090, 2 = Ruminococcaceae u. OTU ID 325, 3 = Faecalibacterium u. OTU ID 2096, 4 = Faecalibacterium u. OTU ID 415, 5 = Lachnospiraceae u. OTU ID 592, 6 = Faecalibacterium u. OTU ID 624, 7 = Faecalibacterium u. OTU ID 168, 8 = Subdoligranulum u. OTU ID 54, 9 = Collinsella u. OTU ID 184, 10 = Faecalibacterium u. OTU ID 2248, 11 = Ruminococcaceae u. OTU ID 156, 12 = *Alistipes shahii* WAL 8301, 13 = Prevotella u. OTU ID 459, 14 = Faecalibacterium u. OTU ID 1488, 15 = Ruminococcaceae u1868, 16 = Lachnospiraceae u. OTU ID 288, 17 = Lachnospiraceae u.OTU ID 355, 18 = Ruminococcaceae u. OTU ID 2088, 19 = *Ruminococcus bromii*, 20 = Anaerotruncus u. OTU ID 1923.
(PNG)

S4 Fig Subject 101 Sequencing and Metabolite Profile. Coprostanol, Cholesterol TIC content and 16S Sequence percent abundance for Subject 101 (Metronidazole treated CDI-subject). Spearman’s correlation coefficient (rho) between 16S sequence abundance and coprostanol also shown.
(PNG)

S5 Fig Subject 102 Sequencing and Metabolite Profile. Coprostanol, Cholesterol TIC content and 16S Sequence percent abundance for Subject 102 (Metronidazole treated CDI-subject). Spearman’s correlation coefficient (rho) between 16S sequence abundance and coprostanol also shown.
(PNG)

S6 Fig Subject 103 Sequencing and Metabolite Profile. Coprostanol, Cholesterol TIC content and 16S Sequence percent abundance for Subject 103 (Metronidazole treated CDI-subject). Spearman’s correlation coefficient (rho) between 16S sequence abundance and coprostanol also shown.
(PNG)

S7 Fig Subject 104 Sequencing and Metabolite Profile. Coprostanol, Cholesterol TIC content and 16S Sequence percent abundance for Subject 104 (Metronidazole treated CDI-subject). Spearman’s correlation coefficient (rho) between 16S sequence abundance and coprostanol also shown.
(PNG)

S8 Fig Subject 301 Sequencing and Metabolite Profile. Coprostanol, Cholesterol TIC content and 16S Sequence percent abundance for Subject 301 (Vancomycin treated CDI-subject). Spearman’s correlation coefficient (rho) between 16S sequence abundance and coprostanol also shown.
(PNG)

S9 Fig Subject 302 Sequencing and Metabolite Profile. Coprostanol, Cholesterol TIC content and 16S Sequence percent abundance for Subject 302 (Vancomycin treated CDI-subject). Spearman’s correlation coefficient (rho) between 16S sequence abundance and coprostanol also shown.
(PNG)

S10 Fig Subject 303 Sequencing and Metabolite Profile. Coprostanol, Cholesterol TIC content and 16S Sequence percent abundance for Subject 303 (Vancomycin treated CDI-subject). Spearman’s correlation coefficient (rho) between 16S sequence abundance and coprostanol also shown.
(PNG)

S11 Fig Subject 501 Sequencing and Metabolite Profile. Coprostanol, Cholesterol TIC content and 16S Sequence percent abundance for Subject 501 (Healthy subject with 90 days prior antibiotic exposure). Spearman’s correlation coefficient (rho) between 16S sequence abundance and coprostanol also shown.
(PNG)

S12 Fig Subject 502 Sequencing and Metabolite Profile. Coprostanol, Cholesterol TIC content and 16S Sequence percent abundance for Subject 502 (Healthy subject with 90 days prior antibiotic exposure). Spearman’s correlation coefficient (rho) between 16S sequence abundance and coprostanol also shown.
(PNG)

S13 Fig Subject 503 Sequencing and Metabolite Profile. Coprostanol, Cholesterol TIC content and 16S Sequence percent abundance for Subject 503 (Healthy subject with 90 days prior antibiotic exposure). Spearman’s correlation coefficient (rho) between 16S sequence abundance and coprostanol also shown.
(PNG)

S14 Fig Subject 701 Sequencing and Metabolite Profile. Coprostanol, Cholesterol TIC content and 16S Sequence percent abundance for Subject 701 (Healthy control subject. Spearman’s correlation coefficient (rho) between 16S sequence abundance and coprostanol also shown.
(PNG)

S15 Fig Subject 702 Sequencing and Metabolite Profile. Coprostanol, Cholesterol TIC content and 16S Sequence percent abundance for Subject 702 (Healthy control subject. Spearman’s correlation coefficient (rho) between 16S sequence abundance and coprostanol also shown.
(PNG)

S16 Fig Subject 703 Sequencing and Metabolite Profile. Coprostanol, Cholesterol TIC content and 16S Sequence percent abundance for Subject 703 (Healthy control subject. Spearman’s correlation coefficient (rho) between 16S sequence abundance and coprostanol also shown.
(PNG)

S1 Table Reference OTU (refOTU) Identification number and corresponding phylogeny based on Silva database used in this study. (XLSX)
