# Supplementary figures and images for: An Integrated Metabolomic and Microbiome Analysis Identified Specific Gut Microbiota Associated with Fecal Cholesterol and Coprostanol in Clostridium difficile Infection
Source: PLoS One. 2016 Feb 12;11(2):e0148824. doi: 10.1371/journal.pone.0148824 (PMC4752508; doi:10.1371/journal.pone.0148824)

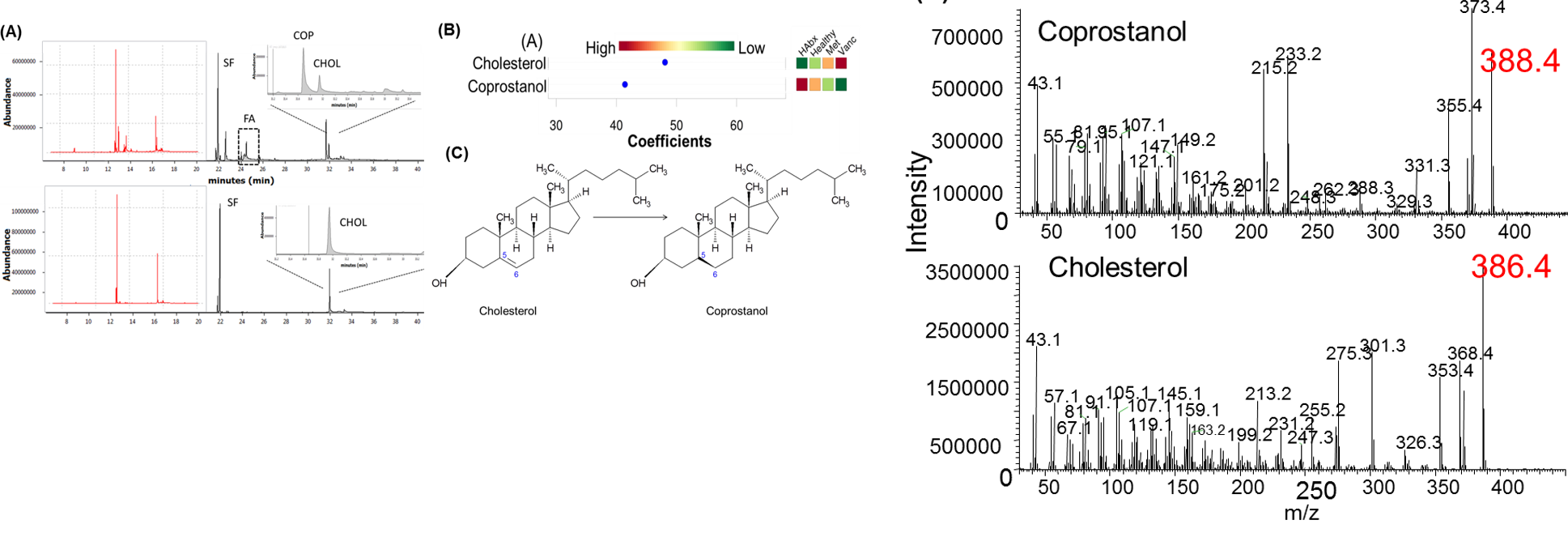

Supplement: S1 Fig — (A) Representative GC-chromatogram of typical healthy subject time point (above) and CDI subject time point (below). SF is the solvent front and FA represents fatty acid peaks that consistently eluted in the time window but whose identity and features could not be discriminated. (B) Weighted sum of partial least square regression coefficients for cholesterol and coprostanol with their inverse abundance for each of the four cohorts examined in this study (C) Chemical structure of cholesterol and coprostanol that were discriminating features between CDI and healthy longitudinal fecal samples. Right hand panel: Fragmentation pattern coprostanol and cholesterol with leading M+ ion (m/z) shown in red. (PNG) [file pone.0148824.s001.png]

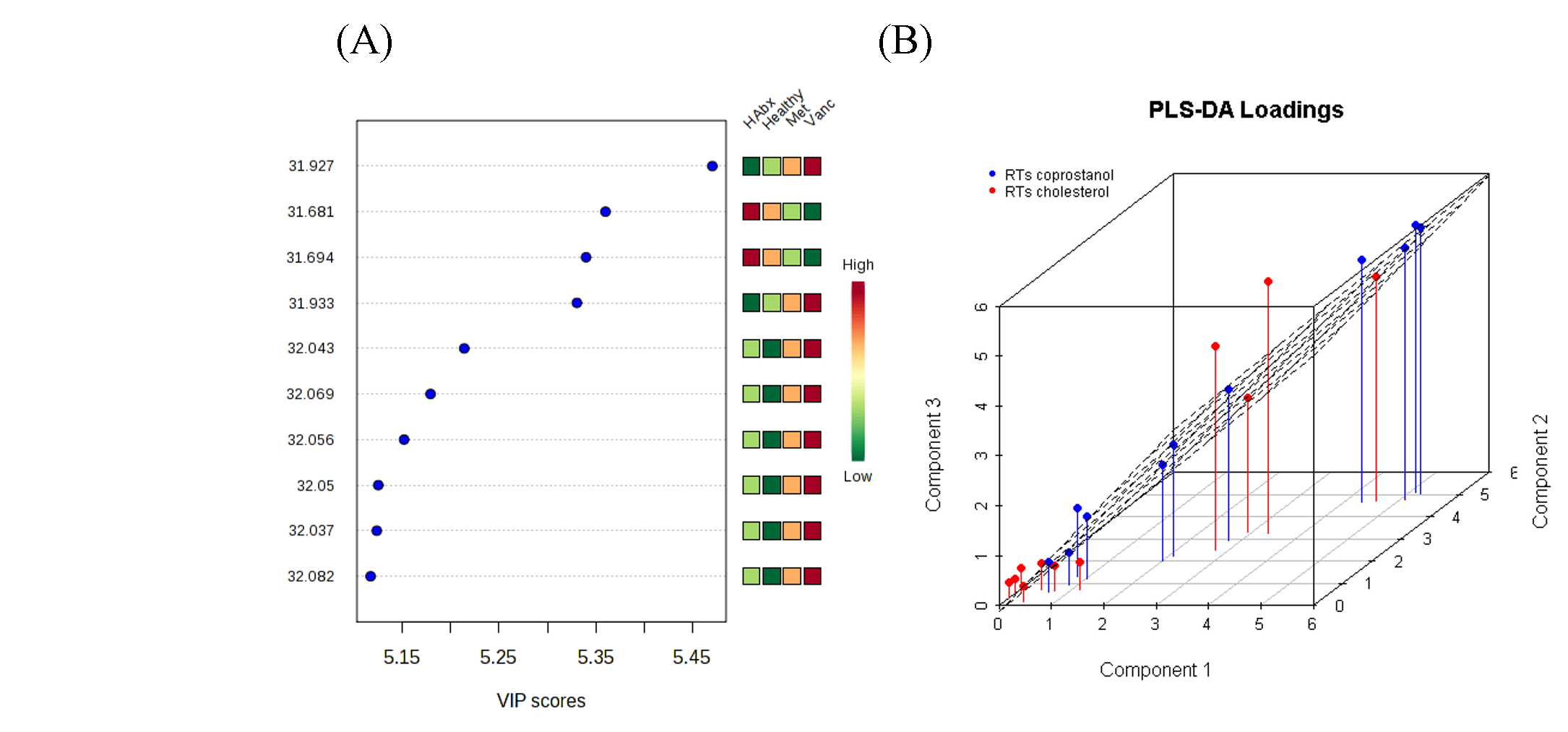

Supplement: S2 Fig — As an approach to identify compounds of CDI and Healthy, differential RTs were sought after. (A) PLS-DA VIP scores of the ten highest rank retention times with their relative abundances in healthy subjects with antibiotic history (HAbx), Healthy subjects with no reported antibiotic history (Healthy), and CDI subjects taking Metronidazole (Met) and Vancomycin (Vanc). (B) The contribution of the top VIP scores identified as cholesterol and coprostanol to the PLS-DA loadings according to antibiotic exposure and CDI-drug exposure (Healthy vs HAbx vs Met vs Vanc). The dispersion of retention times across the three principal components suggests some Healthy, HAbx, and Metronidazole fecal samples contained both cholesterol and coprostanol during the 90 days of longitudinal analysis. (PNG) [file pone.0148824.s002.png]

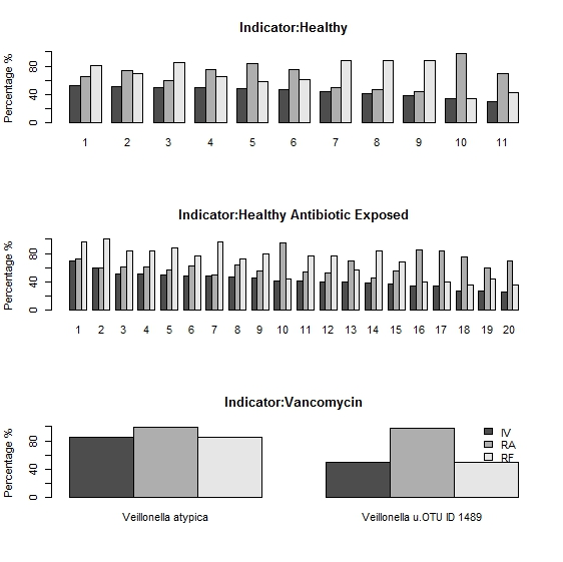

Supplement: S3 Fig — Species related to either high coprostanol or low coprostanol were grouped by subject cohort (Healthy, HAbx, or Vanc). The indicator value (IV), relative abundance (RA), and relative frequency (RF) are shown in vertical axis and are sorted horizontally according to their indicator values. The Met cohort contained no indicator species. Top: 1 = Lachnospiraceae u. OTU ID 1693, 2 = Prevotella u. OTU ID 557, 3 = Faecalibacterium u. OTU ID 798, 4 = Lachnospiraceae u. OTU ID 848, 5 = Lachnospiraceae u. OTU ID 1984, 6 = Faecalibacterium u. OTU ID 1507, 7 = Eubacterium eligens ATCC 27750, 8 = Bacteroides D22, 9 = Bacteroides stercoris ATCC.43183, 10 = Faecalibacterium u. OTU ID 1848, 11 = Coprococcus catus. Middle: 1 = Faecalibacterium u. OTU ID 2090, 2 = Ruminococcaceae u. OTU ID 325, 3 = Faecalibacterium u. OTU ID 2096, 4 = Faecalibacterium u. OTU ID 415, 5 = Lachnospiraceae u. OTU ID 592, 6 = Faecalibacterium u. OTU ID 624, 7 = Faecalibacterium u. OTU ID 168, 8 = Subdoligranulum u. OTU ID 54, 9 = Collinsella u. OTU ID 184, 10 = Faecalibacterium u. OTU ID 2248, 11 = Ruminococcaceae u. OTU ID 156, 12 = Alistipes shahii WAL 8301, 13 = Prevotella u. OTU ID 459, 14 = Faecalibacterium u. OTU ID 1488, 15 = Ruminococcaceae u1868, 16 = Lachnospiraceae u. OTU ID 288, 17 = Lachnospiraceae u.OTU ID 355, 18 = Ruminococcaceae u. OTU ID 2088, 19 = Ruminococcus bromii, 20 = Anaerotruncus u. OTU ID 1923. (PNG) [file pone.0148824.s003.png]

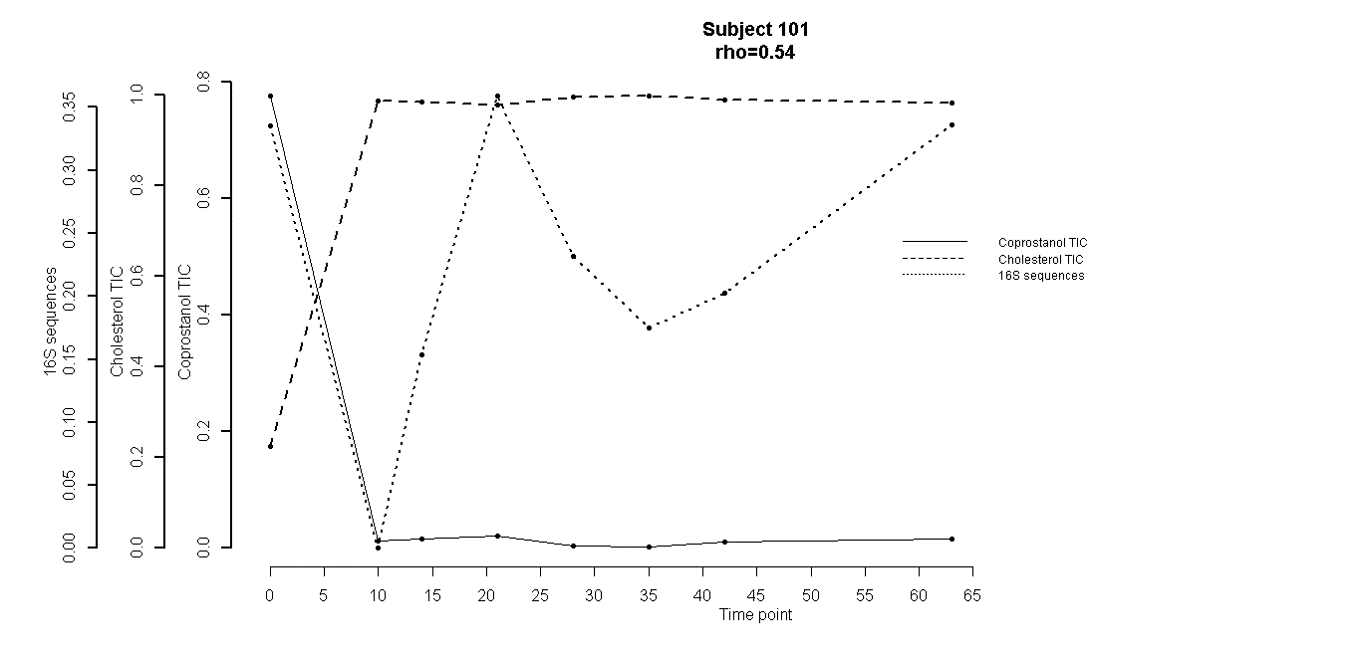

Supplement: S4 Fig — Coprostanol, Cholesterol TIC content and 16S Sequence percent abundance for Subject 101 (Metronidazole treated CDI-subject). Spearman’s correlation coefficient (rho) between 16S sequence abundance and coprostanol also shown. (PNG) [file pone.0148824.s004.png]

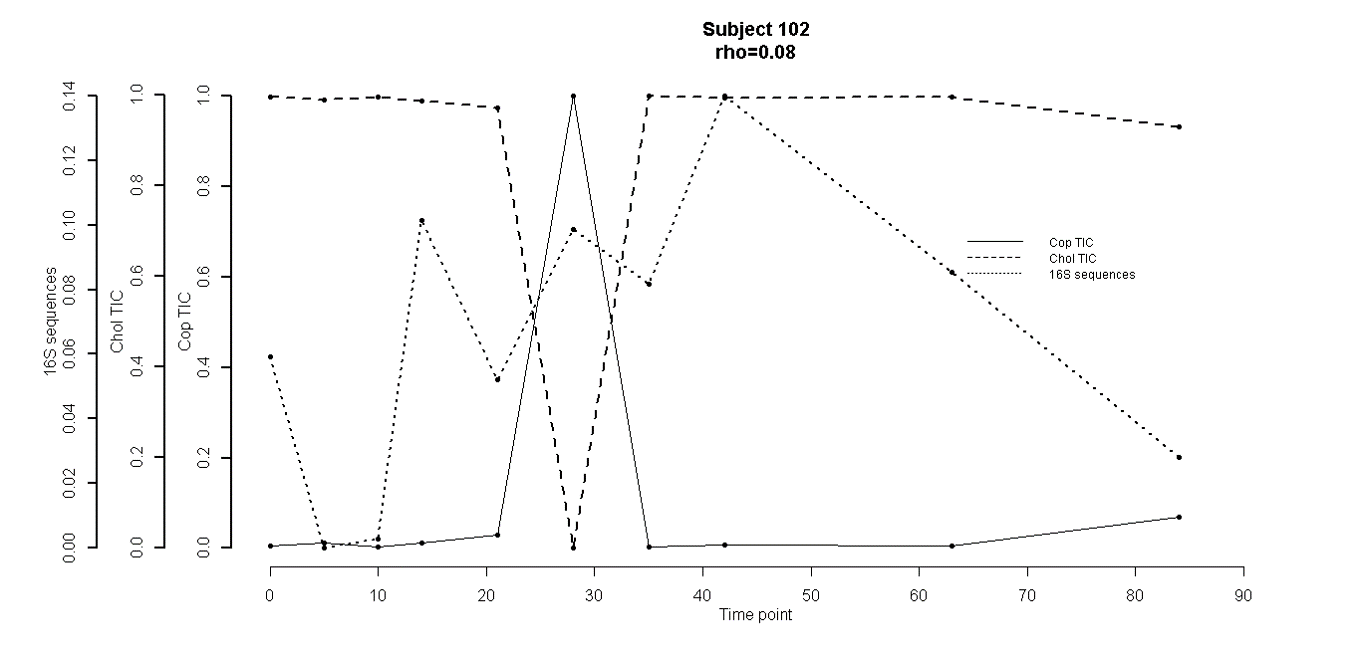

Supplement: S5 Fig — Coprostanol, Cholesterol TIC content and 16S Sequence percent abundance for Subject 102 (Metronidazole treated CDI-subject). Spearman’s correlation coefficient (rho) between 16S sequence abundance and coprostanol also shown. (PNG) [file pone.0148824.s005.png]

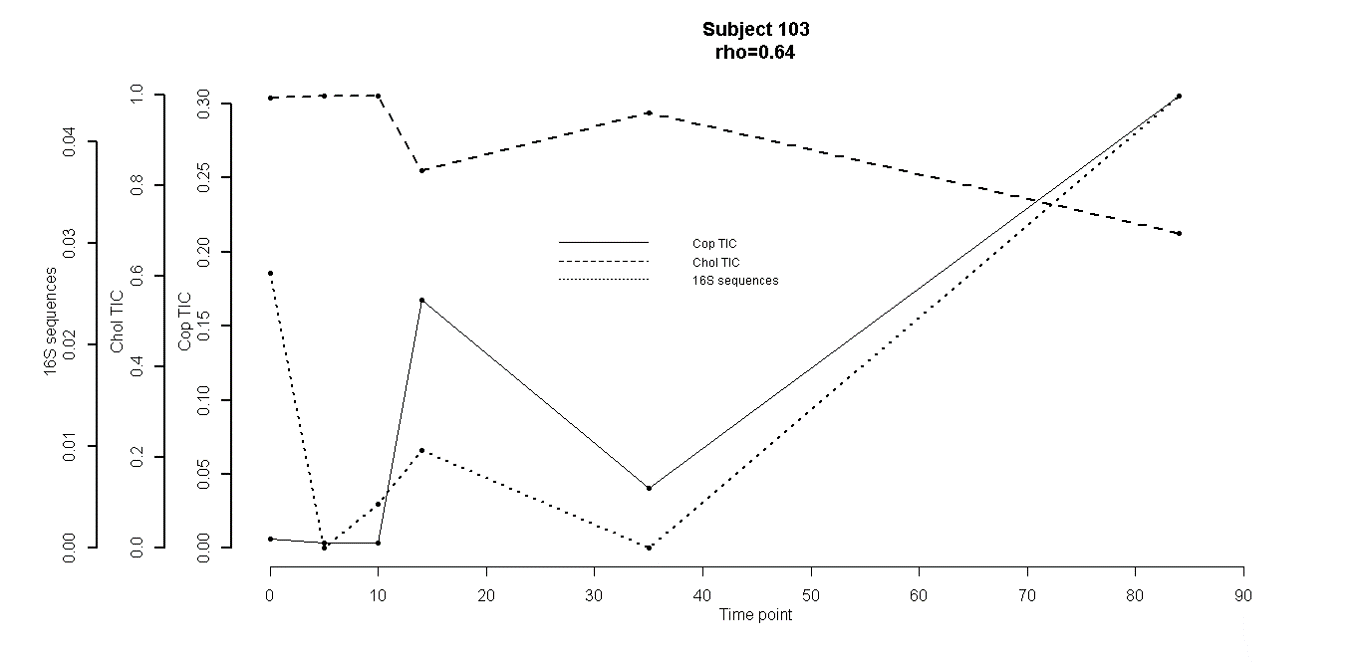

Supplement: S6 Fig — Coprostanol, Cholesterol TIC content and 16S Sequence percent abundance for Subject 103 (Metronidazole treated CDI-subject). Spearman’s correlation coefficient (rho) between 16S sequence abundance and coprostanol also shown. (PNG) [file pone.0148824.s006.png]

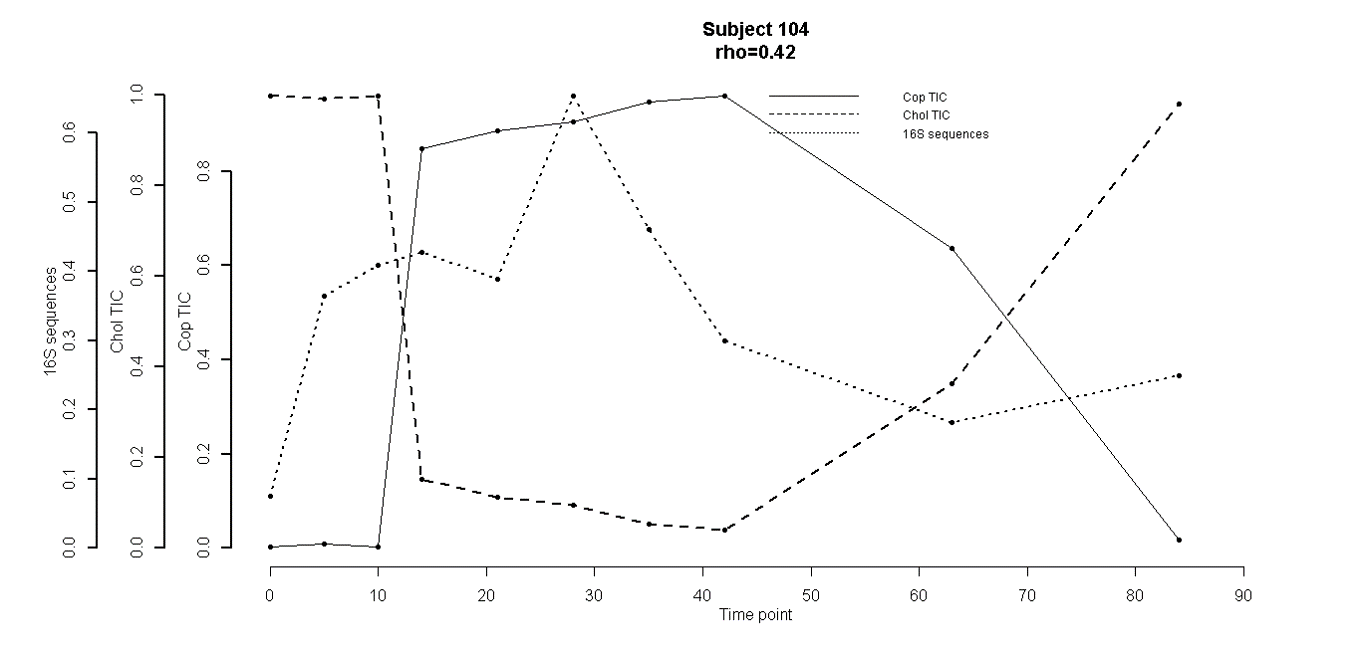

Supplement: S7 Fig — Coprostanol, Cholesterol TIC content and 16S Sequence percent abundance for Subject 104 (Metronidazole treated CDI-subject). Spearman’s correlation coefficient (rho) between 16S sequence abundance and coprostanol also shown. (PNG) [file pone.0148824.s007.png]

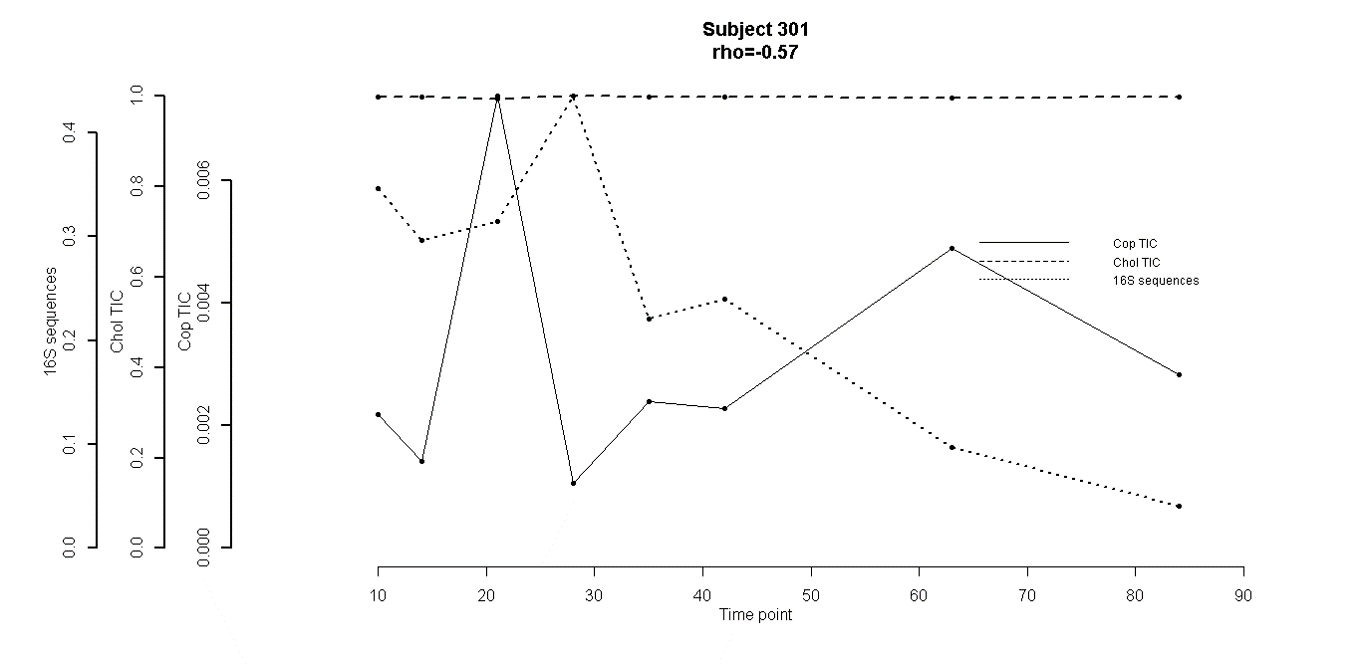

Supplement: S8 Fig — Coprostanol, Cholesterol TIC content and 16S Sequence percent abundance for Subject 301 (Vancomycin treated CDI-subject). Spearman’s correlation coefficient (rho) between 16S sequence abundance and coprostanol also shown. (PNG) [file pone.0148824.s008.png]

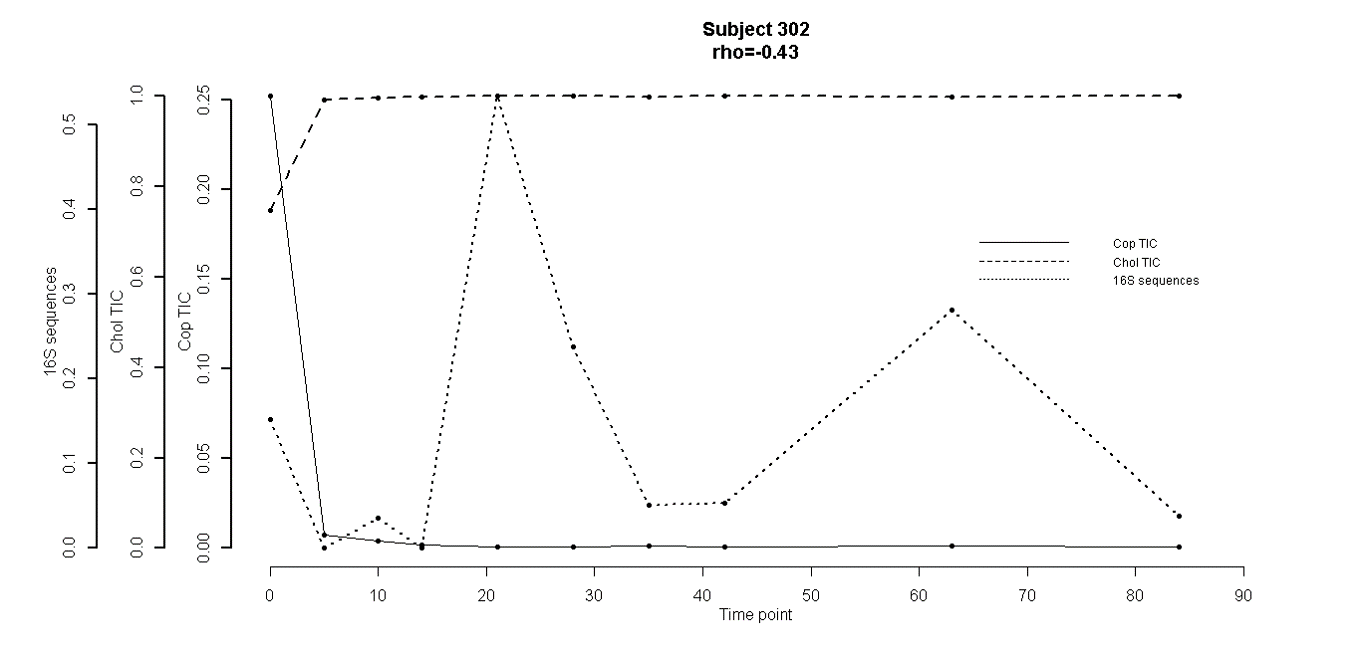

Supplement: S9 Fig — Coprostanol, Cholesterol TIC content and 16S Sequence percent abundance for Subject 302 (Vancomycin treated CDI-subject). Spearman’s correlation coefficient (rho) between 16S sequence abundance and coprostanol also shown. (PNG) [file pone.0148824.s009.png]

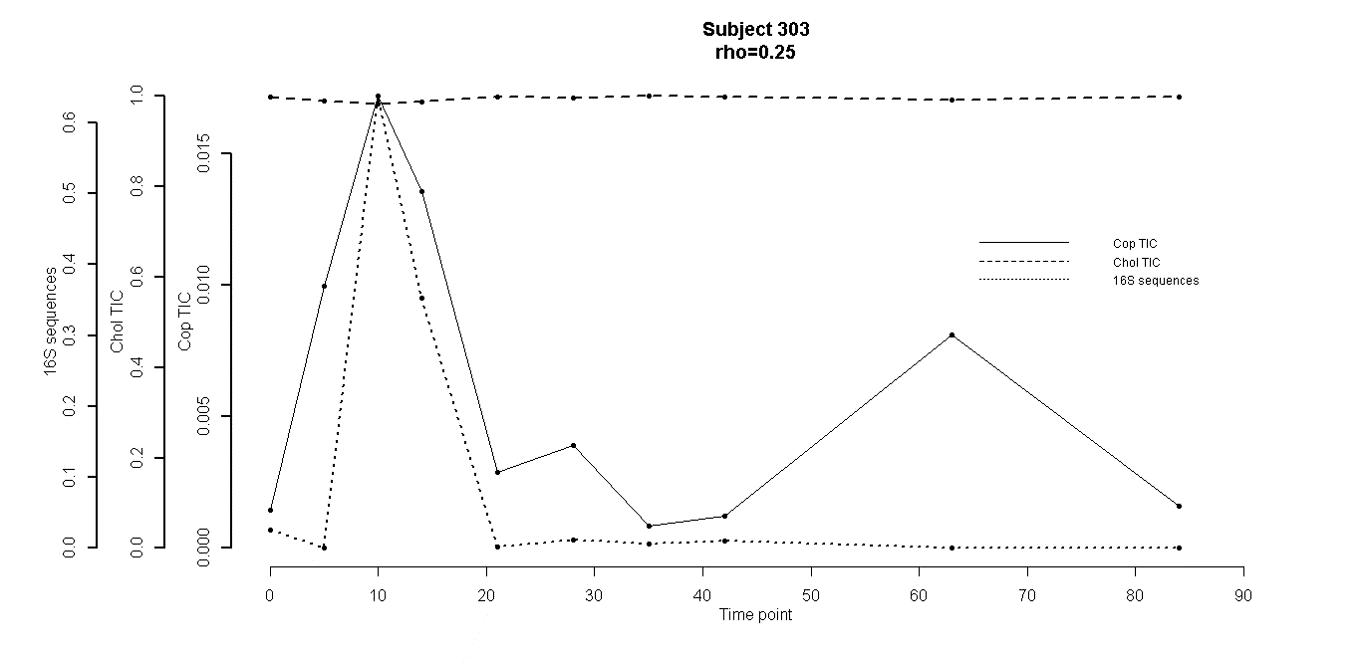

Supplement: S10 Fig — Coprostanol, Cholesterol TIC content and 16S Sequence percent abundance for Subject 303 (Vancomycin treated CDI-subject). Spearman’s correlation coefficient (rho) between 16S sequence abundance and coprostanol also shown. (PNG) [file pone.0148824.s010.png]

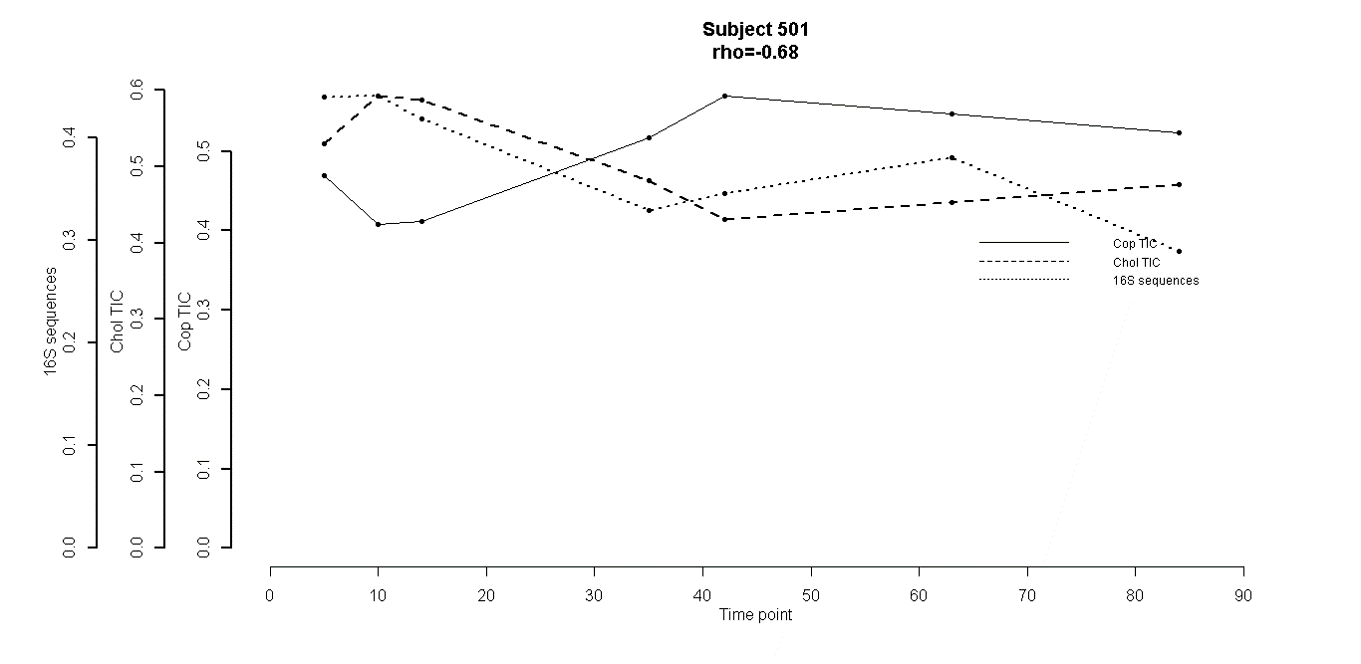

Supplement: S11 Fig — Coprostanol, Cholesterol TIC content and 16S Sequence percent abundance for Subject 501 (Healthy subject with 90 days prior antibiotic exposure). Spearman’s correlation coefficient (rho) between 16S sequence abundance and coprostanol also shown. (PNG) [file pone.0148824.s011.png]

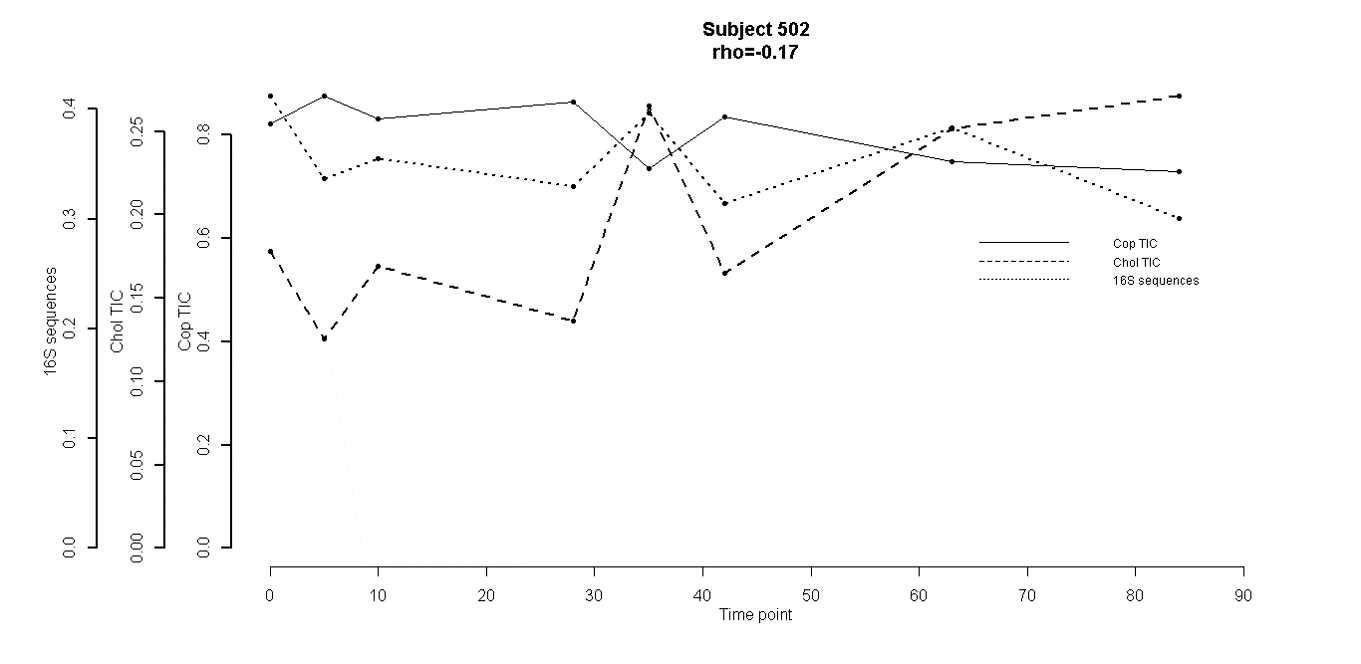

Supplement: S12 Fig — Coprostanol, Cholesterol TIC content and 16S Sequence percent abundance for Subject 502 (Healthy subject with 90 days prior antibiotic exposure). Spearman’s correlation coefficient (rho) between 16S sequence abundance and coprostanol also shown. (PNG) [file pone.0148824.s012.png]

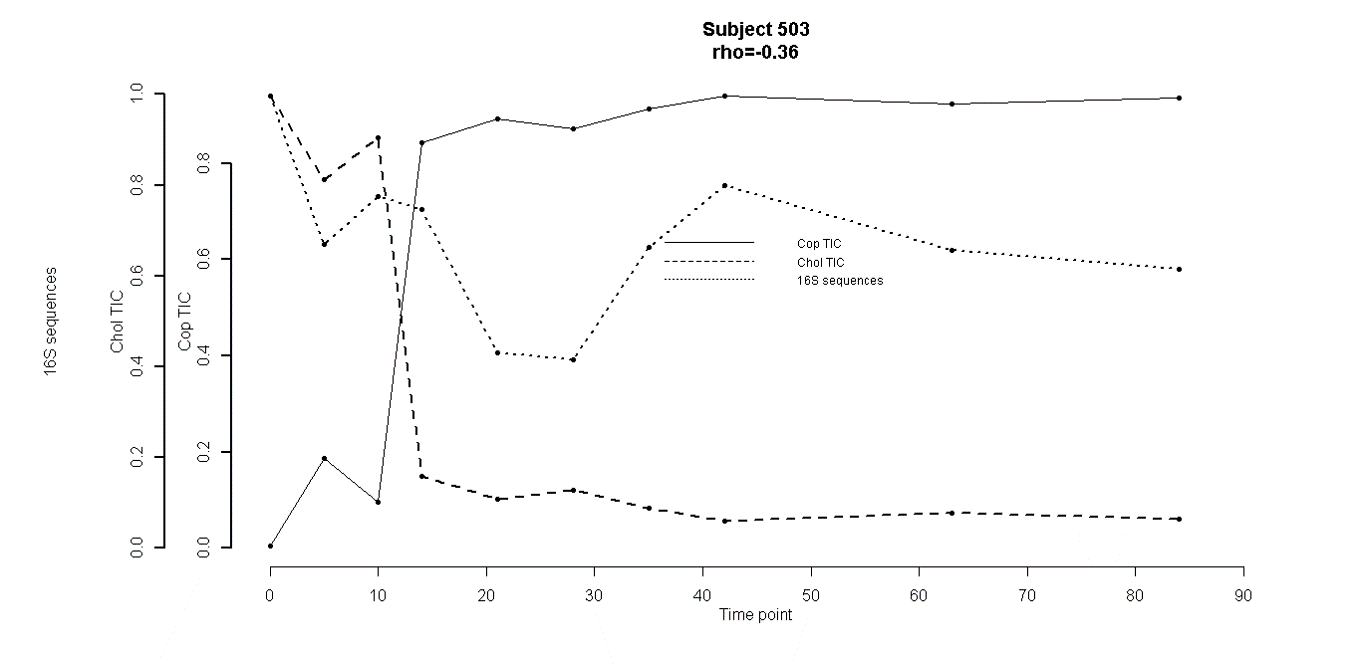

Supplement: S13 Fig — Coprostanol, Cholesterol TIC content and 16S Sequence percent abundance for Subject 503 (Healthy subject with 90 days prior antibiotic exposure). Spearman’s correlation coefficient (rho) between 16S sequence abundance and coprostanol also shown. (PNG) [file pone.0148824.s013.png]

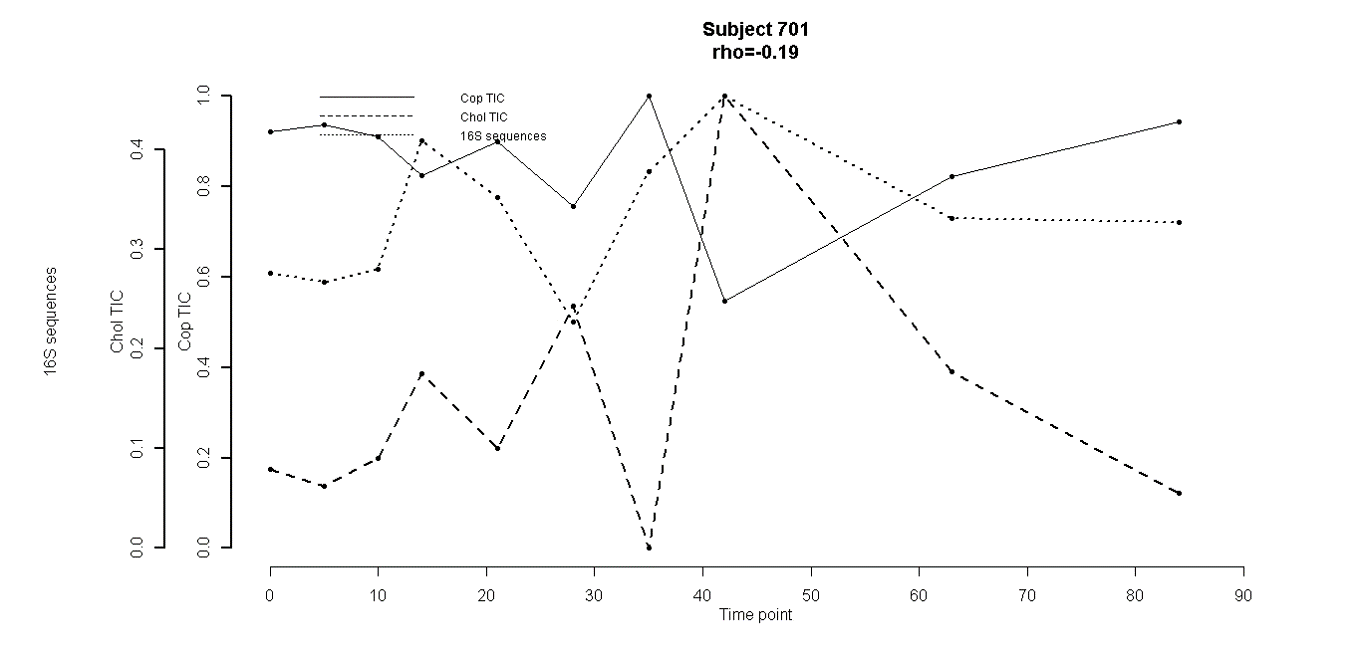

Supplement: S14 Fig — Coprostanol, Cholesterol TIC content and 16S Sequence percent abundance for Subject 701 (Healthy control subject. Spearman’s correlation coefficient (rho) between 16S sequence abundance and coprostanol also shown. (PNG) [file pone.0148824.s014.png]

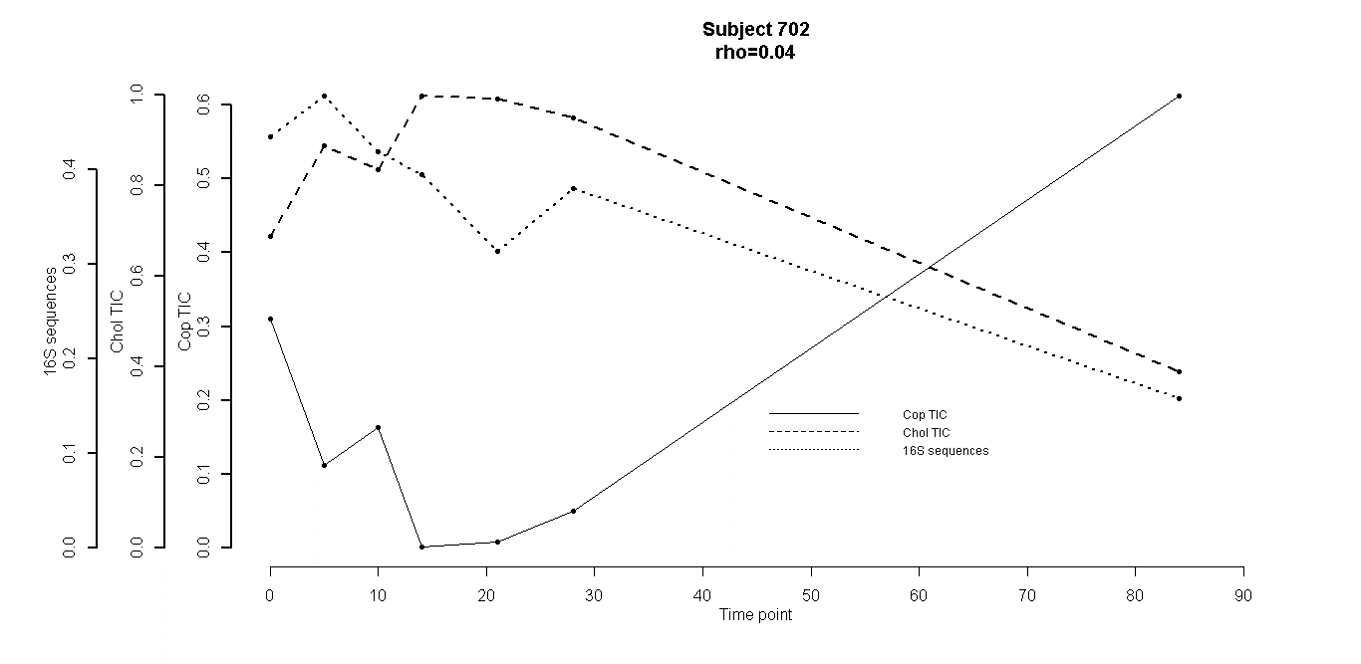

Supplement: S15 Fig — Coprostanol, Cholesterol TIC content and 16S Sequence percent abundance for Subject 702 (Healthy control subject. Spearman’s correlation coefficient (rho) between 16S sequence abundance and coprostanol also shown. (PNG) [file pone.0148824.s015.png]

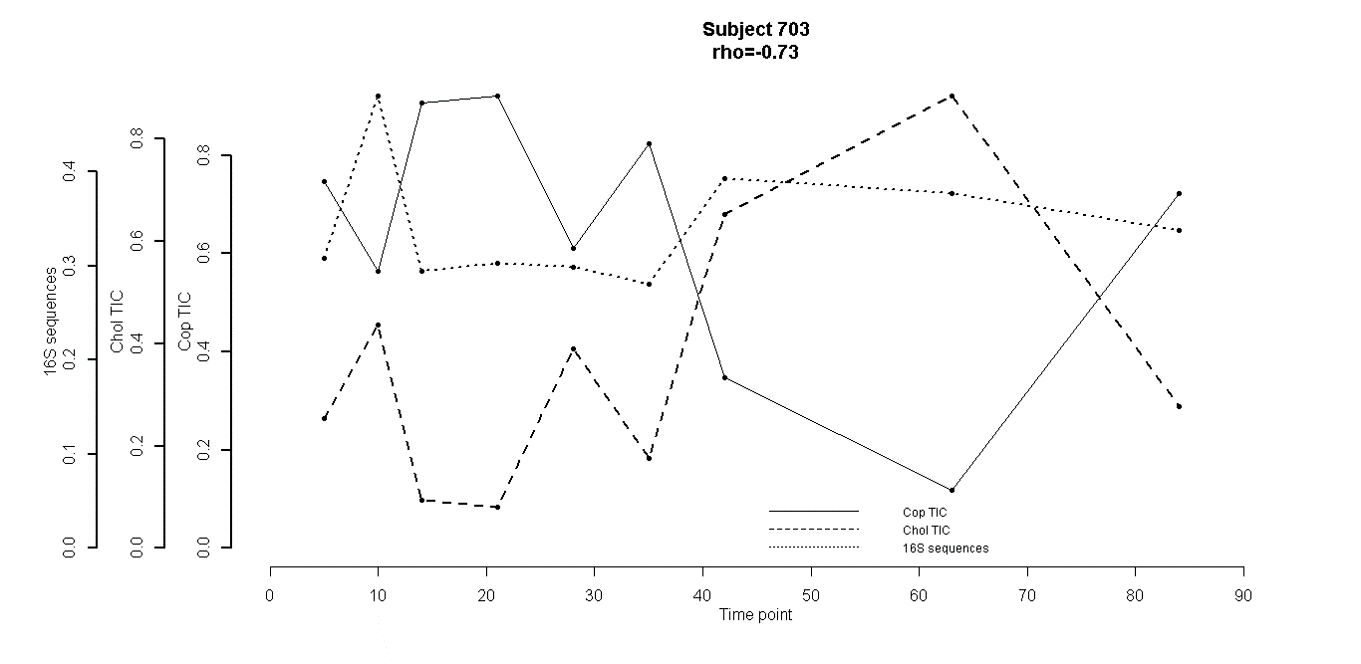

Supplement: S16 Fig — Coprostanol, Cholesterol TIC content and 16S Sequence percent abundance for Subject 703 (Healthy control subject. Spearman’s correlation coefficient (rho) between 16S sequence abundance and coprostanol also shown. (PNG) [file pone.0148824.s016.png]
